# Visual processing during natural reading

**DOI:** 10.1038/srep26902

**Published:** 2016-05-27

**Authors:** Béla Weiss, Balázs Knakker, Zoltán Vidnyánszky

**Affiliations:** 1 Brain Imaging Centre, Research Centre for Natural Sciences, Hungarian Academy of Sciences, Budapest 1117, Hungary; 2 Faculty of Information Technology and Bionics, Pázmány Péter Catholic University, Budapest 1083, Hungary; 3 Department of Cognitive Science, Budapest University of Technology and Economics, Budapest 1111, Hungary

## Abstract

Reading is a unique human ability that plays a pivotal role in the development and functioning of our modern society. However, its neural basis remains poorly understood since previous research was focused on reading words with fixed gaze. Here we developed a methodological framework for single-trial analysis of fixation onset-related EEG activity (FOREA) that enabled us to investigate visual information processing during natural reading. To reveal the effect of reading skills on orthographic processing during natural reading, we measured how altering the configural properties of the written text by modifying inter-letter spacing affects FOREA. We found that orthographic processing is reflected in FOREA in three consecutive time windows (120–175 ms, 230–265 ms, 345–380 ms after fixation onset) and the magnitude of FOREA effects in the two later time intervals showed a close association with the participants’ reading speed: FOREA effects were larger in fast than in slow readers. Furthermore, these expertise-driven configural effects were clearly dissociable from the FOREA signatures of visual perceptual processes engaged to handle the increased crowding (155–220 ms) as a result of decreasing letter spacing. Our findings revealed that with increased reading skills orthographic processing becomes more sensitive to the configural properties of the written text.

Reading is a unique human ability that plays a pivotal role in the development and functioning of our modern society^1^,^2^. In spite of this, our knowledge regarding the neural basis of reading remains limited. This is because natural reading is essentially an active sensory-motor process^3^,^4^,^5^ where visual sampling of the orthographic information is subserved by consecutive saccadic eye movements, yet, most previous research on reading^6^,^7^,^8^,^9^,^10^,^11^,^12^ impose strict gaze stability to control the time course of visual information processing and to avoid signal artefacts that eye movements generate^13^,^14^,^15^. Although the passive presentation approach has revealed crucial properties of orthographic processing, it fails to capture the active nature of reading and thus might obscure some key aspects of neural processes subserving active sampling of visual information during natural reading.

Reading skills develop gradually from childhood to adolescence^2^,^16^ and acquiring reading expertise has an overall effect on visual information processing^17^,^18^. Previous research investigating word reading with fixed gaze revealed that neural coding of letters and words is subserved by ventral occipito-temporal visual cortical regions^6^,^10^,^11^, primarily the letter-form and word-form areas, where letter-form and word-form selective neural responses emerge starting from around 150 ms and 220 ms after stimulus presentation, respectively^19^. Furthermore, it was also shown that selectivity of both functional magnetic resonance imaging (fMRI) responses in the visual word-form area^2^,^16^ and early event-related potential (ERP) components^20^,^21^,^22^ to written letter and word stimuli increases in skilled readers as compared to beginners or individuals with reading deficits. However, to what extent the outlined scheme of neural processing of orthographic information also holds true in the case of natural reading has remained an open question. This is because previous studies that were aiming at investigating neural processes of natural reading were focusing on methodological issues^13^,^23^,^24^ (such as feasibility of concurrent EEG and eye movement registration during reading and elimination of eye movement artefacts) and on higher-level lexical/semantic effects^13^,^23^,^24^,^25^,^26^ rather than on orthographic processing.

Importantly, when aiming at uncovering the neural substrates of orthographic processing in natural reading, the approach commonly taken in the research on word reading with fixed gaze^6^,^10^,^11^,^19^ – e.g. comparing neural responses to words with those evoked by pseudowords or other objects – appears to be inappropriate. This is because in the results obtained from the comparison of words and non-word stimuli the neural processes subserving skilled orthographic processing would be confounded by the visual cortical effects of overall differences in sensory-motor processes and task demands. Instead, it seems to be a more adequate approach to manipulate the second-order configural relations^27^ within the written words by changing spatial distances among the letters. Altering inter-letter spacing is known to affect orthographic processing and reading speed without hampering the overall process of natural reading^28^,^29^,^30^. Previous research on object vision has shown that perceptual expertise makes visual processing of the highly trained object category more sensitive to its configural information^27^,^31^,^32^,^33^ and that such expertise-related increase in sensitivity for configural properties was reflected in the ERP component associated with object-selective visual cortical processing^34^,^35^,^36^. In accordance with this, behavioural studies showed that skilled readers are tuned to the standard spacing^37^ and increasing letter spacing to more than 1.5 times the normal spacing leads to reduced reading speed in skilled readers^28^,^30^,^38^. However, the same manipulation has no effect or even increases reading efficiency in children^39^ and dyslexics^38^, respectively. Based on this, we hypothesized that the core components of orthographic processing during natural reading might be revealed by contrasting the fixation onset-related EEG activity during reading text with normal letter spacing to that with altered spacing. We also assumed that there might be a close association between sensitivity to configural properties of orthographic information and reading skills, which in turn would lead to increased letter spacing effects on visual processing during natural reading in fast as compared to slow readers.

Altering letter spacing is expected to affect both the early parallel extraction of letters and computation of bigrams as well as the later stage of word-form processing, and thus could allow us to investigate the neural signatures of these two prominent stages of orthographic processing during natural reading. In particular, based on previous findings showing that object-selective visual cortical responses are enhanced to objects of expertise^16^,^34^,^35^,^36^,^40^,^41^,^42^, we predicted that when reading text with altered letter spacing, the neural responses associated with letter- and word-form-related processing will be diminished when compared to those during reading with normal letter spacing. Furthermore, we also expected to find a close association between the magnitude of the neural response difference between normal and altered spacing and reading skills. To test these assumptions in the current study we contrasted the fixation onset-related EEG activity during reading a text with normal letter spacing to that with increased and reduced spacing in normal readers. We will refer to these results as expertise-driven configural effects, since they reflect those neural processes of visual information processing during natural reading which become selective for normal letter spacing with expertise and will be disturbed independently of whether spacing is increased or decreased, i.e. when configural properties of the written text are modified. In addition, letter spacing also affects the reciprocal interference, i.e. crowding among letters located in close proximity^28^,^43^: overall visual perceptual processing load will be increased with enhanced crowding as a result of decreased letter spacing. Therefore, we identified the neural processes that are associated with the modulation of the overall visual processing load and dissociated them from those involved in expert orthographic processing. We will refer to these results as visual processing load effects.

To address the technical challenges that are posed by natural viewing on analysis of fixation onset-related EEG activity, we developed an experimental framework based on simultaneous recording of eye-tracking and EEG data. For elimination of eye-movement artefacts we applied independent component analysis (ICA) enhanced by eye-tracking information. In order to test the expertise-driven configural and visual processing load effects of letter spacing with a control of potential confounding eye-movement variables, we used hierarchical linear modelling of single-trial EEG activity. We expected letter spacing effects at different stages of orthographic processing. Although topography and timing of reading processes are revealed to some extent, only a limited number of studies is available on the spatio-temporal properties of EEG activity in natural reading conditions. Accordingly, instead of performing statistical evaluation on an arbitrary subset of electrodes and time intervals based on visual inspection of letter spacing trends we performed a data-driven statistical analysis in the spatio-temporal domain by considering all electrodes and time samples in the 0–600 ms time range after the onset of fixations.

## Methods

This section contains the description of the main natural reading experiment. Information about the control experiments can be found in Supplementary Methods.

### Participants

Twenty-four undergraduate students (11 female) participated in the main experiment. Average age was 22.33 years with a standard deviation (SD) of 1.78 years (range: 20–26 years). All participants were right-handed (as assessed by the standard Edinburgh Handedness Inventory^44^) native speakers of Hungarian, reported having typical reading skills and had normal or corrected-to-normal vision. None of them had any history of neurological or psychiatric diseases. The study was approved by the local ethics committee of the Department of Cognitive Science, Budapest University of Technology and Economics and all methods were carried out in accordance with the approved guidelines. Subjects gave written informed consent.

### Visual stimuli and experimental procedure

**Figure 1:**
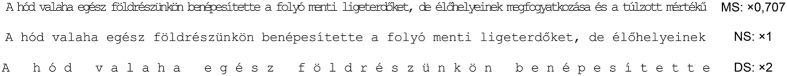
Sample visual stimuli used to reveal the effects of letter spacing on brain activity during natural reading. Hungarian paragraphs were presented line-by-line and subjects were instructed to read the text at their own pace from left to right. During the experiment eye-tracking and EEG data were recorded simultaneously to assess eye movement and brain activity correlates of letter spacing modulation. Three different levels of letter spacing were used: minimal spacing (MS, top row), normal spacing (NS, middle row) and double spacing (DS, bottom row). The size of inter-letter spacing for particular condition was obtained by multiplying the default letter spacing size with the multiplication factor denoted in the figure.

Thirty-two Hungarian texts were used in this experiment. Originally, paragraphs were constructed by the Programme for International Student Assessment (PISA), Organisation for Economic Co-operation and Development (OECD) for assessment of reading literacy of high school students. Text was presented line-by-line using the monospaced 13 pt Courier New font. A 26′′ liquid-crystal display was applied at refresh rate of 60 Hz and by exploiting its central region only with a native resolution of 1600 × 1200 pixels, the remaining left and right borders were kept black during the experiment. The viewing distance was 56 cm. Black letters were used on the central white background having extent of 47 × 35° (degrees of visual angle). Horizontal text lines were centred on the screen. The length of lines was maximized to 44.85° by applying a 2 cm margin on both right and left borders of the white background. However, the actual line extent depended on the particularly warped text portion and the applied letter spacing. Three different levels of letter spacing (LS) were used (Fig. 1): minimal spacing (MS; 0.707 times the normal spacing); normal spacing (NS; the distance between consecutive characters is 1.16 times the width of the lowercase x^28^); double spacing (DS; 2 times the NS). In order to prevent the psycholinguistic variables from confounding the letter spacing experimental factor the order of spacing was set to be random. The randomization was carried out for all subjects before the experiment to assure even distribution of the number of text lines with different letter spacing. Repetition of the same spacing for more than two consecutive lines was not allowed. The width of characters was ∼0.27°, definitely above the critical print size^28^ and accordingly, the spacing size was ∼0.31° in the case of NS. Subjects were instructed to read at their own pace and to switch to the next line by pressing the space button on the keyboard. Before the appearance of text lines a fixation point was presented for 1 s at the beginning of the forthcoming text line. After each paragraph a single sentence test statement was presented and participants had to report with a mouse button press whether it was true (left button) or false (right button). Paragraphs were organized into eight blocks, each block contained four paragraphs. At the end of blocks subjects could choose to have a rest or continue the experiment. In case of rest, re-calibration of the eye-tracker was carried out (see below). Generation of stimuli, control of the experimental procedure and collection of subjects’ responses were performed using custom written scripts and the Psychophysics Toolbox 3^45^,^46^,^47^ under MATLAB R2008a (The MathWorks Inc., Natick, MA, USA).

### Recordings

Eye-tracking data were recorded from the left eye using an iView X™ Hi-Speed 1250 system (SensoMotoric Instruments GmbH, Teltow, Germany). The sampling rate was 1250 Hz. Head stabilization was realized by using the chin rest and the forehead support of the eye-tracking system as well as by applying foam cushions for fixation of the EEG cap and electrodes as needed. Calibration was carried out using the built-in 13 points routine at the beginning of the experiment and after all resting periods. Quality of eye-tracking was controlled by real-time visual inspection of the recordings.

EEG data were acquired using a BrainAmp Standard amplifier with a 64-channel actiCAP active electrode system and the BrainVision Recorder 1.2 software (Brain Products GmbH, Munich, Germany). Sixty-two electrodes were placed according to the 10-10 international standard and one additional electrode was positioned below the right eye. FP1 and FP2 locations were omitted due to the application of a forehead support for head stabilization. All channels were referenced to the right mastoid (TP10), while the ground electrode was at AFz. All electrode impedances were kept below 5 kΩ. Data were sampled at 500 Hz.

For the synchronization of EEG and eye-tracking recordings, the same trigger signals were sent to the EEG system through a standard trigger port and to the eye-tracking system using the Ethernet protocol. Trigger signals coded start and end times of paragraphs and text lines as well as subject responses.

### Processing of eye-tracking data

Eye-tracking data were processed with two procedures: first, to enhance EEG artefact elimination; second, to characterise overall eye movement properties throughout the whole experiment. The first procedure started with semi-automatic pre-processing of eye-tracking data. After automatic detection of eye-blinks, 1 s long artefact-free segments were selected by visual inspection of the recordings. The preferred position of segments was the screen centre in order to minimize the possible effects of stimulus onset^13^, preparation and execution of motor actions for pressing the buttons. Saccade and fixation parameters were obtained from these segments by an adaptive algorithm^48^ with the following settings: max. saccade velocity = 1000°/s; max. saccade acceleration = 100000°/s^2^; min. fixation duration = 40 ms; min. saccade duration = 10 ms; α_AA_ = 0.7; β_AA_ = 0.3; initial saccade peak velocity detection threshold PT_1_ = 50°/s. The main advantage of this novel approach is that it relies on adaptive estimation of threshold values by taking into consideration the local noise level. The selected segments and eye movement parameters were used for EEG artefact elimination. During the second procedure, the same adaptive algorithm with identical parameters was applied on eye-tracking data corresponding to whole text lines for an overall statistical assessment of eye movement properties. Additionally, reading speed was also estimated for all text lines by dividing the number of words in a line by the time required to read the whole text line. Subject-level central tendencies of saccade amplitude (SA), fixation duration (FD) and reading speed were calculated for all spacing conditions separately by taking the median values. More detailed presentation of eye-tracking results is to be available elsewhere. Eye-tracking data processing was performed in MATLAB R2011b (The MathWorks Inc., Natick, MA, USA) with custom scripts based on the toolbox provided by dr. Marcus Nyström.

### EEG processing

EEG signal processing comprised pre-processing of the recordings, extraction and selection of single-trial FOREA data and scalp current density (SCD) transformation (also known as Laplace transformation).

We developed a novel multi-stage pre-processing method based on a standard ICA approach^49^ that was enhanced by eye-tracking information. The main goal of this procedure was the elimination of artefacts related to eye movements^13^,^14^,^15^,^50^,^51^ (saccadic spike potentials originating from muscle activity and artefacts attributable to rotation of the corneo-retinal dipoles), but artefacts of other type were also eliminated. The whole pre-processing procedure can be divided into six stages: 1) filtering and semi-automatic detection and removal of artefactual data segments based on different EEG time-series properties; 2) first IC decomposition on the remaining segments; 3) semi-automatic detection and removal of artefactual data segments based on different IC activation time-series properties; 4) second IC decomposition on the remaining segments; 5) detection of artefactual ICs that were obtained by the second decomposition; 6) elimination of artefacts by subtracting the artefactual ICs from continuous recordings. Stages 1–3 are used for selection of most clean data segments that enter the second ICA in order to obtain as good as possible final decomposition. Applying two IC decompositions is considered to be more reliable since additional artefacts could be detected based on different properties of IC activations that are obtained by the first decomposition. Before the second decomposition no artefact elimination is performed by rejecting ICs. During the first 3 stages artefactual segments are cancelled from further processing and a special care is given meanwhile to prevent elimination of data segments only because of eye movements that are inherent and useful events in case of natural reading. Eye-tracking information is used during the 5^th^ stage to enhance the detection of eye-movement artefacts. Pre-processing started with zero-phase digital filtering of continuous recordings. A 4^th^-order Butterworth band-pass (0.5–70 Hz) filter and a 50 Hz notch filter (quality factor Q = 45) were applied with the *filtfilt*() MATLAB function. In the next step, 1 s long filtered EEG segments were extracted corresponding to the previously selected eye-tracking segments. At this stage, various artefact detection criteria were applied on these segments to detect EEG artefacts except the eye movement-related ones. The following settings were used: abnormal values (lower and upper limits: 80 μV), abnormal trends (max. slope: 50 μV/epoch, R^2^ limit: 0.5), improbable data (single channel limit: 5 SD, all channels limit: 5 SD), abnormal distributions (single channel limit: 5 SD, all channels limit: 5 SD), abnormal spectra (upper limits: [50 25] dB, lower limits: [−50 −100] dB, low frequencies [0 20] Hz, high frequencies: [2 40] Hz). Results were validated by visual inspection. The first IC decomposition (stage 2) was performed on segments marked as artefact-free during the previous procedure. The *runica*() routine of the EEGLAB MATLAB toolbox^52^ was used with the following settings: ‘*extended*’, *1*, ‘*stop*’, *10*^−7^, ‘*maxsteps*’, *1024*. The quality of decomposition was validated by visual inspection of ICs’ properties. Further artefact detection was carried out by assessing activations of the obtained ICs (stage 3). Automatic detection was carried out by finding improbable data (single channel limit: 8 SD, all channels limit: 5 SD) and abnormal distributions (single channel limit: 8 SD, all channels limit: 5 SD) in all ICs, the results were validated by visual inspection of the IC activations. A second ICA decomposition (stage 4) was accomplished on the remaining EEG segments and using the same settings as for the first decomposition. Afterwards, a semi-automatic detection and elimination of artefactual ICs was realized (stage 5). Artefactual ICs were automatically detected using the ADJUST EEGLAB plugin^53^ with default settings, and results were validated by visual inspection of different IC properties. Visual assessment of ICs was enhanced by eye-tracking information obtained with the adaptive algorithm (see above) and included inspection of ICs’ topographic distribution, spectral properties as well as single-trial activation images. Single-trial activations were extracted from the 1 s long segments. The trial time interval was [−150 380] ms and the zero time was determined by saccade offset samples, which allows for the examination of IC activities time-locked to eye-movement events. This is what enhances the detection of eye-movement artefacts in natural viewing conditions. Very similar approaches were developed and validated by other research groups^14^,^24^.

For statistical analyses single-trial FOREA data were obtained by extracting [−250 600] ms long epochs from filtered continuous recordings that were cleaned using the corrected ICA weight matrices from the procedure described above. FOREA trials were time-locked to the onset of fixations that corresponded to the offset samples of current (also called incoming) saccades in this study. The trigger events were obtained by adaptive analysis of eye-tracking data. Trials were excluded from further analyses if they overlapped with the first 650 ms or last 200 ms intervals of text line presentation times or if the amplitude of the current saccade was not in the range of 0.3–10°. Only trials with forward, i.e. left-to-right current saccades were considered. Additional selection of trials was performed based on eye-tracking and filtered EEG data. Trials were rejected if they contained eye blinks or the absolute amplitude of EEG surpassed the threshold value of 80 μV. The number of trials that entered statistical analyses was randomly balanced across the 3 letter spacing conditions. The average number of trials was 1008.12 (SD = 371.11; range: 383–1582). Finally, a single-trial baseline correction was applied by subtracting the mean amplitude value of the baseline interval [−50 0] ms from all samples.

To overcome the shortcomings of using a single monopolar reference electrode, re-referencing to average activity of all channels is applied in most of EEG studies. However, this approach may be not appropriate for free-viewing EEG experiments. Despite thorough artefact elimination residual eye movement-related artefacts may remain. Although these artefacts would occur most likely in frontal channels, they would be smeared even to posterior EEG electrodes by average referencing and this could be especially detrimental in the case of data-driven statistical analysis that was applied in this study. A possible solution could be to omit the contaminated electrodes from calculation of average activity^54^. However, this would be also suboptimal since selection of artefactual channels would have to be tested for all subjects and experimental conditions separately and the interpretation of the results could be circumstantial. Thus, instead of average referencing SCD transformation was applied. The SCD transform can also be considered as a standard EEG referencing technique. It was thoroughly validated and its advantages over scalp potential maps were demonstrated by different research groups^55^,^56^,^57^,^58^,^59^,^60^. SCD transformed data can be considered reference independent, the transformation enhances local activity and suppresses activity with broader spatial extent^55^. Accordingly, SCD transformation can be used for reduction of effects of volume conduction and saccadic potentials^61^,^62^. Nevertheless, it does not replace the solution of the inverse problem and thus the obtained results can be interpreted only in the sensor space. SCD transformation was performed using the CSD Toolbox^57^ which follows the spherical spline-based approach introduced by Perrin *et al*.^63^,^64^. The following settings were used: unit sphere radius, m = 4 (spline flexibility), λ = 10^−5^ (smoothing constant), the maximum degree of Legendre polynomials was set to 10. Statistical analyses were carried out on SCD transformed data that were additionally zero-phase filtered by a 4^th^ order low-pass Butterworth filter with a 35 Hz cut-off frequency.

### Statistical analyses

Effects of letter spacing on eye movement measures and reading speed were tested by non-parametric Friedman analysis of variance (ANOVA), since violations of the normality assumption were revealed by the Shapiro-Wilk W test. Post hoc testing of saccade amplitude, fixation duration and reading speed measures between MS and NS as well as between NS and DS conditions was carried out by the Wilcoxon matched pairs test with Bonferroni correction. The relationship between reading speed in the NS and the other two conditions was tested by correlation analysis. Skipped Spearman’s correlation coefficients (r_S_) were calculated with the Robust Correlation Toolbox^65^ by applying an adjusted box-plot rule for outlier detection and by using 1000 iterations to obtain the bootstrap confidence intervals (CI). Levels of significance and confidence were corrected according to the total number (N = 2) of tested correlations (p_Cor_ < 0.05, 97.5% CI). To test the difference of reading speed between MS and NS conditions, the time course of reading speed was evaluated by comparing the median reading speed of MS and NS conditions with the Wilcoxon matched pairs test in 10 temporally consecutive data bins (Supplementary Fig. S1). Additionally, to validate the reading speed results of the main natural reading experiment, a standard behavioural one-minute reading experiment was performed on a different sample of subjects. The detailed description of the control experiment and corresponding methods is available in Supplementary Methods, Control experiment 1.

Hierarchical linear modelling^66^ of FOREA was performed in order to reveal the neural signatures of expertise-driven configural and visual processing load effects while controlling for eye-tracking variables that could also contribute to the variance of brain activity at single-trial level. A very similar approach was previously used to assess the time course of visual word recognition in an ERP experiment^8^. At subject level we applied two different models on single-trial FOREA data. Effects of visual processing load were tested by a multiple linear regression (MLR) model in which letter spacing was considered a continuous variable, while expertise-driven configural effects were assessed with an analysis of covariance (ANCOVA) model using letter spacing as a categorical variable and by setting a −1 2 −1 contrast for the MS, NS and DS levels of letter spacing (ANCOVA_LSCONT_). To control for effects of eye-tracking covariates, 6 eye-tracking measures (current, first preceding and first following saccade amplitudes and fixation durations) were included in both MLR and ANCOVA models, taking into account the multicollinearity between these variables. Hence, this approach allowed for the assessment of pure letter spacing effects by regressing out the potential effects of saccade amplitude and fixation duration covariates. The β coefficients of hierarchical linear modelling analysis will be denoted by subscripts that are generated as follows: the type of the linear model applied at subject level (MLR or ANCOVA) is provided before the hyphen; variables (LS – letter spacing, SA – saccade amplitude, FD – fixation duration) are indicated by the first two letters after the hyphen; the remaining letters denote the previously defined ANCOVA_LSCONT_ contrast (CONT) or mark the time information of eye-movement variables (current (C), first preceding (FP) and first following (FF) saccade amplitudes and fixation durations). The full list of β coefficients is as follows: β_MLR-LS_, β_MLR-SAC_, β_MLR-FDC_, β_MLR-SAFP_, β_MLR-FDFP_, β_MLR-SAFF_, β_MLR-FDFF_, β_ANCOVA-LSCONT_, β_ANCOVA-SAC_, β_ANCOVA-FDC_, β_ANCOVA-SAFP_, β_ANCOVA-FDFP_, β_ANCOVA-SAFF_, β_ANCOVA-FDFF_. For example, expertise-driven configural and visual processing load effects are reflected by the β_ANCOVA-LSCONT_ and β_MLR-LS_ coefficients, respectively, and β_MLR-SAC_ stands for the effects of current saccade amplitudes that are obtained by the multiple linear regression model. Meaning of all β coefficients is provided in the Supplementary Table S1. Trials with missing values were excluded from analysis. Percent of trials with missing values (PTMV) was calculated for all subjects and conditions separately and the following statistics were obtained: min_PTMV_ = 1.75%, max_PTMV_ = 21.84%, mean_PTMV_ = 7.31%, SD_PTMV_ = 4.05%. The maximum of maximal differences of PTMV between the conditions was 7.26%, indicating that the reduction of the number of trials was similar across conditions. The minimum number of remaining trials across all conditions was 926.08 on average (SD = 353.43, range: 366–1498). MLR and ANCOVA β coefficients were calculated for all spatio-temporal samples separately. Group-level effects for letter spacing, saccade amplitude and fixation duration variables were estimated by entering the subject-level β coefficients to group-level analysis. A one-sample t-test was carried out on the β coefficients using the cluster-based permutation testing framework^67^,^68^ in the spatio-temporal domain for multiple comparisons correction. In the case of EEG data the cluster-based permutation testing can be considered more appropriate compared to other correction methods since it takes the neighbourhood information also into account in time and spatial (and even spectral) domains. In order to increase the sensitivity of statistical analyses, β coefficient maps were pre-processed with threshold-free cluster enhancement (TFCE)^69^,^70^. The number of bootstrap repetitions was 1000 and default settings (E = 0.5, H = 2, dh = 0.1) were used for TFCE. The neighbourhood matrix of EEG electrodes was generated by the *limo*_*get*_*channeighbstructmat*() function with a neighbourhood distance parameter set to 0.5. During statistical evaluation, the minimum number of neighbouring channels was 2. Additionally, to reveal the effects with moderate spatial extent, a 1D cluster-based permutation test was also applied at group level by performing clustering only along the time dimension (1D clustering). To keep the presentation and interpretation of the results as simple as possible, we will consider the results of spatio-temporal clustering by default, and refer to the results obtained with 1D clustering only where necessary. The significance threshold was set to p_Cluster_ < 0.05. The hierarchical linear modelling analysis was performed using custom-written MATLAB scripts and the LIMO^66^ EEGLAB plugin that also incorporates several tools from the FieldTrip toolbox^71^.

The relationship between reading skill and the effects of letter spacing on EEG activity was tested by correlating the average reading speed RS_AVG_ with subject-level β_MLR-LS_ and β_ANCOVA-LSCONT_ coefficients. Reading speed was averaged across the 3 letter spacing conditions to obtain an overall reading skill measure at subject level. This was feasible since very strong positive significant correlations were found between reading speeds in different letter spacing conditions. The subject-level β coefficients were also averaged in predefined spatio-temporal clusters based on significant group-level results. For this purpose, 3 channel clusters were considered: left occipito-temporal channel cluster (LOTC; channels P5, P7, PO7, PO9), right occipito-temporal channel cluster (ROTC; channels P6, P8, PO8, PO10) and parietal channel cluster (PC; channels Pz, P1, P2, POz). Time intervals for averaging were defined as the intersections of significant time intervals of the selected channels and were determined for early, middle and late significant effects separately. Six correlations were calculated for the expertise-driven configural effects reflected by β_ANCOVA-LSCONT_ (LOTC, ROTC and PC in the 125–155 ms time range; ROTC in the 240–260 ms time interval; LOTC and PC in the 355–380 ms time range), while only 2 correlations were considered for the visual processing load effects based on β_MLR-LS_ coefficients (ROTC and PC channel clusters in the 170–220 ms time interval). Correlation coefficients were calculated with the same settings as in the case of reading speed analysis, except for the levels of significance and confidence, that were corrected according to the total number (N = 8) of tested correlations (p_Cor_ < 0.05, 99.375% CI).

Finally, to compare the effects revealed during natural reading with results that could be obtained in more standard ERP experimental conditions, the potential effects of letter spacing on EEG activity were also tested during fixed-gaze word reading. The detailed description of the control experiment and corresponding methods is available in Supplementary Methods, Control experiment 2.

## Results

The structure of the Results section is organized in a way to provide a justification of the applied methods before the presentation and validation of the main findings. In case of active vision experiments, a thorough assessment of behavioural measures has to be carried out before the final selection of EEG analysis since eye-movement variables may confound the experimental factors and this can lead to misinterpretation of results. Accordingly, a detailed presentation of eye-tracking and reading speed results will be provided in the first subsection. After getting acquainted with the trends of saccade amplitude and fixation duration measures, the potential effects of these eye-movement variables on fixation onset-related EEG activity will be outlined at the beginning of the second subsection. The potential effects of covariates are considered during the selection of statistical methods and an overview of EEG statistical analysis is provided before the description of main results. Finally, in the last subsection, letter spacing effects obtained in the natural reading condition are compared to results of fixed-gaze word reading and references to further validations are given.

### The effect of letter spacing on eye movements and reading speed

Modulation of different text properties may influence the eye movements during natural reading. Effects of text features on eye movements are of special interest per se, but are also important during analysis of fixation onset-related EEG activity due to the confounding nature of eye movement features. To address this potential issue, we tested how the modulation of letter spacing affects eye movements and reading speed during natural reading and we found significant effects of letter spacing for both eye movements properties and reading speed. Moreover, in order to determine an appropriate measure of participants’ reading skill that will enter further correlation analyses, we also evaluated the relationship between the reading speeds of different letter spacing conditions.

**Figure 2:**
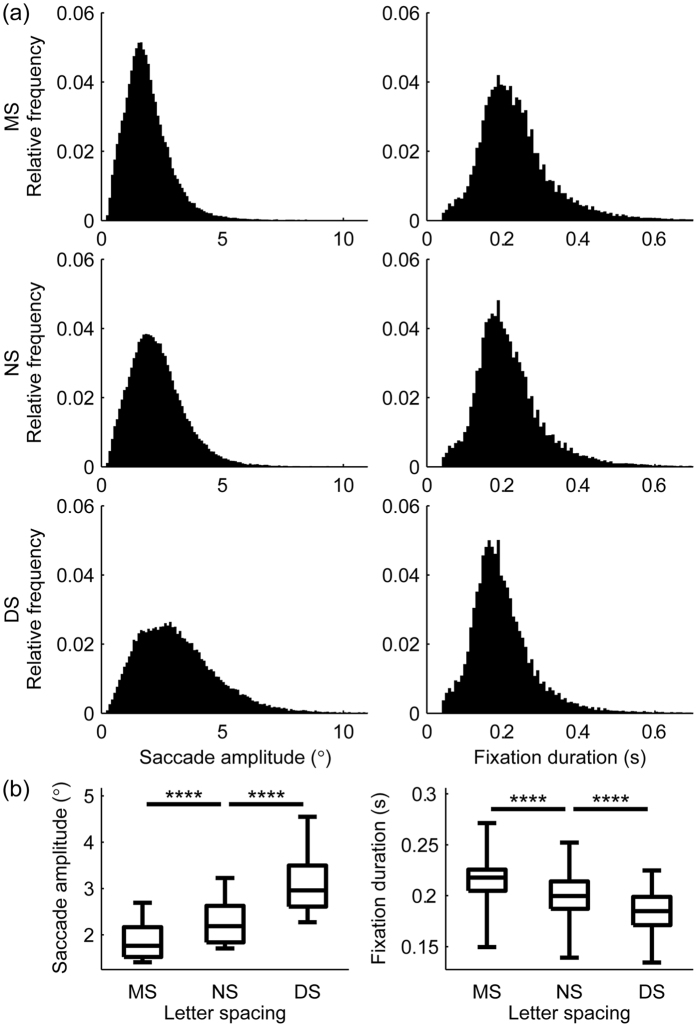
Eye movement correlates of letter spacing modulation in natural reading. Panel (**a**) shows the group-level relative frequency histograms of saccade amplitude (left) and fixation duration (right) measures for the 3 letter spacing conditions (MS, NS and DS) using pooled data from all subjects. Panel (**b**) shows box and whisker plots for saccade amplitude and fixation duration based on individual median values. Whiskers represent minima and maxima. Significant main effect of letter spacing was obtained for both measures, the horizontal lines marked by asterisks denote significance levels of post hoc tests (****p_Bonferroni_ < 0.0001). Saccade amplitudes are considered in degrees of visual angle (°).

**Figure 3:**
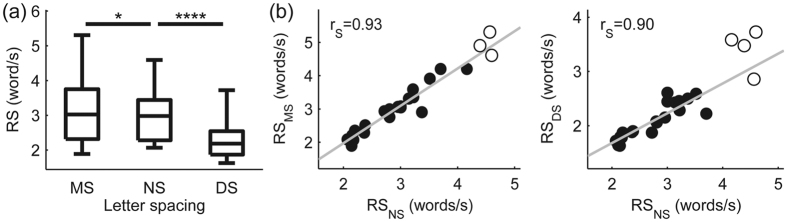
Reading speed results obtained for the natural reading experiment. Panel (**a**) shows box and whisker plots of reading speed (RS) for the 3 letter spacing conditions (MS, NS and DS) based on individual median values. Whiskers represent minima and maxima. It was found that letter spacing has a significant effect on reading speed, the horizontal lines marked by asterisks denote significance levels of post hoc tests (*p_Bonferroni_ < 0.05; ****p_Bonferroni_ < 0.0001). Scatter plots in panel (**b**) present the relationship of reading speed between the NS and the other two letter spacing conditions (MS and DS). Significant (p_Cor_ < 0.05) positive correlations were obtained for both scatter plots. Grey lines denote best fit lines, open circles mark bivariate outliers detected by the adjusted box-plot rule and r_S_ stands for the Spearman’s correlation coefficient.

First, we estimated how changing letter spacing alters eye movements. Significant main effects of letter spacing were obtained for both fixation duration (χ^2^(2, 24) = 44.33, p < 0.00001) and saccade amplitude (χ^2^(2, 24) = 48, p < 0.00001) eye movement measures (see Fig. 2). Fixation duration was reduced with the increase of letter spacing (post hoc results: FD_MS_ > FD_NS_ and FD_NS_ > FD_DS_, p_Bonferroni_ < 0.0001), suggesting that visual processing load is decreased as a result of increased inter-letter spacing. On the other hand, saccade amplitudes were increased with larger letter spacing (post hoc results: SA_MS_ < SA_NS_ and SA_NS_ < SA_DS_, p_Bonferroni_ < 0.0001), which is in agreement with previous results^26^,^28^,^35^. Letter spacing also had a significant effect on reading speed (χ^2^(2, 24) = 39, p < 0.00001). Reading was fastest at the smallest inter-letter distance and decreased with the increase of letter spacing (Fig. 3 panel (a), post hoc results: RS_MS_ > RS_NS_, p_Bonferroni_ < 0.05; RS_NS_ > RS_DS_, p_Bonferroni_ < 0.0001). Finally, very strong significant (p_Cor_ < 0.05) positive correlations were found between the reading speed in the normal spacing and the other 2 conditions (Fig. 3, panel (b); RS_NS_ vs. RS_MS_: r_S_ = 0.93, 97.5% bootstrap confidence interval CI = [0.69 0.99], number of detected outliers NO = 3); RS_NS_ vs. RS_DS_: r_S_ = 0.90, 97.5% CI = [0.63 0.98], NO = 4). These results indicate that subjects reading faster in the normal spacing condition outperform slower readers in the other two conditions as well, and accordingly, general reading skill can be characterized by reading speed averaged across different letter spacing conditions (RS_AVG_).

The small but significant increase of reading speed in the MS condition as compared to the NS was an unexpected finding, since previous research found decrease in reading speed when letter spacing was reduced below the NS^28^,^30^,^38^. However, a closer look at how reading speed changed during the experiment revealed that reading speed was similar in the minimal and normal letter spacing conditions at the beginning, and reading became faster in the MS condition starting from the middle part of the experiment (Supplementary Fig. S1). This suggests that larger reading speed in the minimal as compared to the normal letter spacing condition might be explained by practice effects. This conclusion is also supported by a control experiment (see Supplementary Methods, Control experiment 1) in which we tested the effect of letter spacing on reading speed in a one-minute silent reading task using the same stimuli as in the main experiment. We found a non-significant trend of decreased reading speed in the case of minimal compared to normal spacing (Supplementary Fig. S2 panel (a)), which is consistent with the previous findings^28^,^30^,^38^. In agreement with the main experiment, the one-minute reading control experiment revealed strong positive correlations between the reading speed in the NS and the other 2 conditions with modified letter spacing (Supplementary Fig. S2 panel (b)). In this control experiment we also included a vertical reading condition with normal letter spacing. However, we found only a non-significant trend when correlation analysis was performed for reading speed in the vertical and horizontal conditions with normal letter spacing. These results suggest that pervasive modulations of configural properties of written text, such as text rotation, may affect orthographic processing and reading speed more significantly by hindering the overall process of natural reading.

### EEG results of natural reading

**Figure 4:**
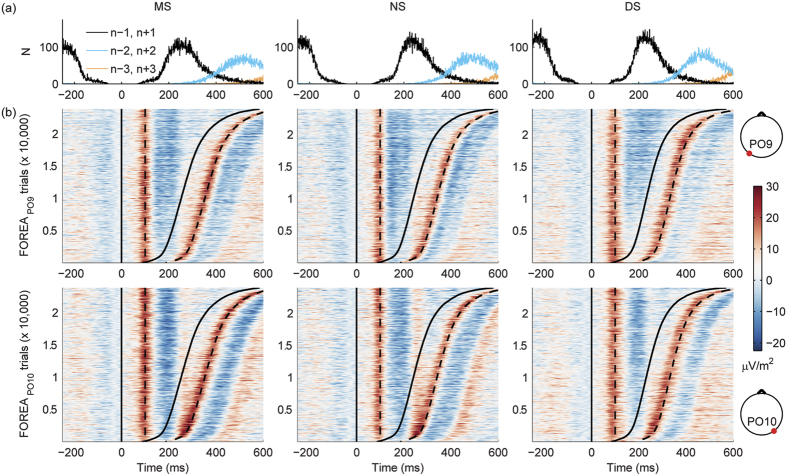
Single-trial EEG activity triggered to the onset of fixations. Panel (**a**) shows histograms of onset times for first preceding and following (n − 1, n + 1), second preceding and following (n − 2, n + 2) and third preceding and following (n − 3, n + 3) events considered for triggering of EEG data. It can be seen that the number of 2^nd^ and 3^rd^ preceding trigger events (n − 2, n − 3) is very close or equal to zero in the [−250 0) ms time interval, and within the first 600 ms after the onset of current fixations there is only a limited number of third following trigger events (n + 3). Besides the amplitude of current saccades and the duration of current fixations the early fixation onset-related EEG activity (FOREA) may be affected mostly by first preceding (n − 1) and following (n + 1) events and thus one should also control for the eye-movement covariates of these events. To obtain the histograms same trials were used as in panel (**b**). Panel (**b**) presents single-trial images of FOREA for PO9 (upper row) and PO10 (lower row) electrodes. Trials were sorted according to the onset times of first following trigger events and smoothed vertically with a moving average across 50 adjacent trials. FOREA trials were obtained from cleaned and scalp current density transformed EEG data. Black curves denote onset times of current and first following trigger events (solid) and time instances 100 ms after these (dotted). Columns correspond to the 3 letter spacing conditions (MS, NS and DS).

Our eye-tracking results showing a significant effect of letter spacing on both saccade amplitudes and fixation durations imply that the standard ERP trial averaging method might not be appropriate for the analysis of FOREA. This is because it was shown that the amplitude of the incoming saccade has an effect on the amplitude of early EEG activity^13^. Furthermore, averaged FOREA will also be affected by the duration of the fixations, which determines the onset of the subsequent fixations and thus may modulate the later part of FOREA related to current fixation. This is clearly demonstrated by Fig. 4, showing the single-trial images of FOREA with trials sorted according to the onset time of the first subsequent fixations. If properties of eye movements are not affected by experimental factors, trends of FOREA can be evaluated to some extent by matching the trials across the levels of experimental factors according to the eye-movement variables^72^. The only advantage of this approach relies in its simplicity, after the selection of appropriate trials it can be considered as a standard ERP analysis method. On the other side, in addition to the aforementioned assumption it has several other limitations. It is not plausible for testing the effects of overlapping trials and it is cumbersome if several eye-movement variables or continuous experimental factors have to be taken into consideration. Since letter spacing significantly affects saccade amplitude and fixation duration properties of eye movements during natural reading, the trial-matching algorithm is not feasible for testing the letter spacing effects on FOREA. Therefore, we developed an alternative approach for single-trial analyses of FOREA based on hierarchical linear modelling and applied the trial-matching method only for validation of specific trends. Using the alternative approach, various linear models can be applied at the subject level to reveal the effects of experimental factors on FOREA and importantly, potential effects of covariates can also be handled within this framework. In this study, to assess the expertise-driven configural and visual processing load effects, ANCOVA and MLR models were applied, respectively. In the ANCOVA model, expertise-driven configural was quantified by the ANCOVA_LSCONT_ contrast over letter spacing, which was regarded as a categorical variable, while in the MLR model letter spacing was applied as a continuous variable that also directly reflected the visual processing load effect. Current, first preceding and first following fixation durations and saccade amplitudes were included as covariates into both linear models in order to exclude the possibility that the obtained expertise-driven configural and visual processing load effects are confounded by the changes in these eye movement parameters as a result of letter spacing manipulation. Sample design matrices can be found in Supplementary Fig. S3. For both linear models β coefficients were calculated at all channel × time samples and the individual β coefficients were evaluated at the group level by a one-sample t-test. Multiple comparisons correction was carried out by spatio-temporal cluster-based permutation testing by default, and additionally, to reveal potential effects of moderate spatial extent, a temporal cluster-based correction (1D clustering) was also applied. However, in this case, only channels and time intervals of special interest were considered based on spatio-temporal correction results.

Using the single-trial analysis approach significant effects of letter spacing on FOREA were revealed. Significant expertise-driven configural effects were found in 3 consecutive time windows (120–175 ms, 230–265 ms and 345–380 ms after the onset of fixations), and these effects were clearly dissociable from the significant visual processing load effects that occurred in the 155–220 ms time interval. By evaluating the relationship between the magnitude of FOREA effects and participants’ reading skill significant correlations were obtained between the latter 2 expertise-driven configural FOREA effects and the reading speed. The correlation results indicate larger FOREA affects for fast as compared to slow readers.

**Figure 5:**
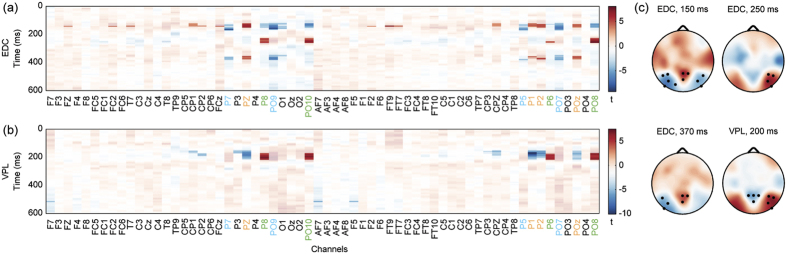
Group-level statistical results of expertise-driven configural and visual processing load effects of letter spacing modulation on brain activity during natural reading. The figure presents spatio-temporal distributions of group-level t-values for expertise-driven configural (EDC) and visual processing load (VPL) effects (panels (**a**,**b**)) as well as topographic distributions of t-values for time instances of special interest (panel (**c**)). In panels (**a**,**b**) significant (p_Cluster_ < 0.05) t-values are fully opaque, while the opacity was reduced for the rest of the samples. In panel (**c**) significant (p_Cluster_ < 0.05) t-values are denoted by marking the corresponding electrodes. The colour scales used in the topographic plots of EDC and VPL t-values (panel (**c**)) correspond to the colour bars provided in panels (**a**) and (**b**), respectively. Significant (p_Cluster_ < 0.05) EDC effects were found in left occipito-temporal, right occipito-temporal and parietal regions in 3 time intervals: 120–175 ms (in all the three regions), 230–265 ms (in the right occipito-temporal region) and 345–380 ms (in the left occipito-temporal and parietal regions). Significant (p_Cluster_ < 0.05) VPL effects appeared in right occipito-temporal and parietal regions within the 155–220 ms time window. Channels denoted by coloured labels (panels (**a**,**b**)) were assigned to channel clusters for further analysis (blue, left occipito-temporal channel cluster: P5, P7, PO7, PO9; orange, parietal channel cluster: P1, P2, Pz POz; green, right occipito-temporal channel cluster: P6, P8, PO8, PO10). More detailed presentation of temporal properties of significant EDC and VPL effects in these regions can be found in Supplementary Fig. S4. To control for potential effects of eye-tracking measures on FOREA, current, first preceding and first following fixation duration and saccade amplitude covariates were also included into the subject-level ANCOVA and MLR models. The obtained covariate effects were almost identical for the 2 models. Detailed results for current fixation duration and current saccade amplitude regressors are presented in Supplementary Fig. S5.

To reveal how modifying the configural properties of written text affects expertise-driven orthographic processing in natural reading, we contrasted the FOREA during reading a text with normal letter spacing to that with increased and reduced spacing (Fig. 5 panels (a,c)). The earliest significant (p_Cluster_ < 0.05) clusters appeared in the 120–175 ms time range, the cluster with largest spatio-temporal extent was found in the left occipito-temporal region (channels PO9, PO7, P7, P5 and O1). Negative t-values within this cluster indicate more negative FOREA values for the NS condition compared to MS and DS conditions. A significant (p_Cluster_ < 0.05) negative cluster also appeared in the right occipito-temporal region, but with lower t-values and smaller spatio-temporal extent (125–155 ms time range; channels PO10, PO8, P8). The early significant (p_Cluster_ < 0.05) positive cluster with highest t-values was found in the centro-parietal region (Pz, P1, P2, POz, CP1, CPz, CP2) within the 120–155 ms time interval. Significant (p_Cluster_ < 0.05) positive effects with moderate spatio-temporal extent and lower t-values also appeared in Fz, F2, FC2, FCz channels as well as in a small fronto-temporal cluster (channels FT9, FT7 and T7) within a narrow time interval (140–150 ms). These early configural effects were followed by a significant (p_Cluster_ < 0.05) right occipito-temporal positive cluster (channels PO8, PO10, P8 and P6) in the 230–265 ms time range. The topographic distribution of t-statistics in this time interval indicates positive values also for the left occipito-temporal region, however, significant (p_Cluster_ < 0.05) effects were found only on electrode PO9 by using 1D clustering (see Supplementary Fig. S4). The latest significant (p_Cluster_ < 0.05) effects occurred in the 345–380 ms time interval: a negative cluster was found in the left occipito-temporal region (channels PO9, PO7, P7, P5, O1; 345–380 ms time range), while a positive cluster appeared in the parietal zone including channels Pz, P1, P2 and POz (355–380 ms). During this time interval, significant (p_Cluster_ < 0.05) negative t-values in the right occipito-temporal region (channels PO10, P8 and P6) were found by using 1D clustering only. Taken together, the results suggest that altering configural properties of the written text by modifying letter spacing affects visual processing during natural reading in three consecutive time windows: 120–175 ms, 230–265 ms and 345–380 ms.

**Figure 6:**
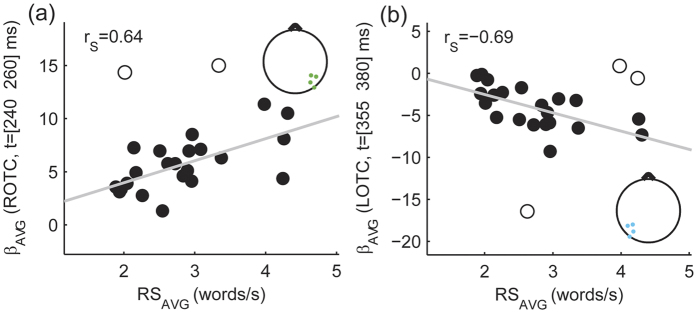
Relationship between reading skill and expertise-driven configural effects of letter spacing modulation on brain activity during natural reading. Scatter plots visualize the relationship between the average reading speed (RS_AVG_) and subject-level expertise-driven configural β_ANCOVA-LSCONT_ coefficients averaged across different spatio-temporal clusters (β_AVG_) that were defined based on group-level results (see Fig. 5 and Supplementary Fig. S4). Significant (p_Cor_ < 0.05) correlations were found between RS_AVG_ and expertise-driven configural effects with intermediate latency (240–260 ms) in the right occipito-temporal region (ROTC: channels P6, P8, PO8, PO10; panel (**a**)) as well as between RS_AVG_ and late (355–380 ms) expertise-driven configural effects in the left occipito-temporal region (LOTC: P5, P7, PO7, PO9 channels; panel (**b**)). Grey lines denote best fit lines, open circles mark bivariate outliers detected by the adjusted box-plot rule and r_S_ stands for the Spearman’s correlation coefficient. Figure insets provide the location of EEG electrodes belonging to ROTC (panel (**a**)) and LOTC (panel (**b**)) channel clusters.

To test for the relationship between the obtained EEG signatures of expertise-driven configural processing during natural reading and the participants’ individual reading skill, we performed a correlation analysis. Significant correlations (p_Cor_ < 0.05) were found between reading speed and expertise-driven configural β coefficients averaged in the following spatio-temporal ranges: right occipito-temporal channel cluster (ROTC) and 240–260 ms time interval (Fig. 6 panel (a), r_S_ = 0.64, 99.375% CI = [0.09 0.90], NO = 2); left occipito-temporal channel cluster (LOTC) and 355–380 ms time interval (Fig. 6 panel (b), r_S_ = −0.69, 99.375% CI = [−0.91 −0.16], NO = 3). No significant correlations were found for other combinations of topographic locations and time ranges.

Next, we used the MLR model to investigate the neural processes that are associated with the modulation of the overall visual processing load as a result of altering letter spacing. The results revealed significant (p_Cluster_ < 0.05) visual processing load effects on FOREA measured on electrode clusters over the occipito-temporal and parietal cortices (Fig. 5 panels (b,c)). A significant (p_Cluster_ < 0.05) positive cluster was revealed in the 170–220 ms time window following the onset of fixations in the right occipito-temporal region including channels P6, P8, PO8 and PO10. This effect peaked on channels P8 and PO10 around 200 ms. Similarly, positive t-values appeared in the left occipito-temporal region, however, the level of significance (p_Cluster_ < 0.05) was reached for channels P7, PO7 and PO9 in the case of 1D clustering only (Supplementary Fig. S4). The positive t-values (that also reflect positive β regression coefficients) for these occipito-temporal electrodes in this particular time interval indicate that the decrease of letter spacing is associated with more negative-going FOREA amplitudes. Furthermore, a significant (p_Cluster_ < 0.05) negative cluster was found in the 155–220 ms time interval in the centro-parieto-occipital region (channels CP1, CP2, P3, Pz, CP3, CPz, P1, P2 and POz) with the strongest effect on channel P1 around 170 ms. These negative t-values denote increase of positive FOREA amplitudes with the decrease of letter spacing. Taken together, these results revealed that increased visual processing load obtained by decreased letter spacing is reflected in right-hemisphere dominant occipito-temporal and parietal increase of EEG activity between 155–220 ms after fixation onset. We found no significant correlation between this EEG signature of visual processing load and participants’ individual reading skill.

### The effect of letter spacing on EEG responses during word reading with fixed gaze

**Figure 7:**
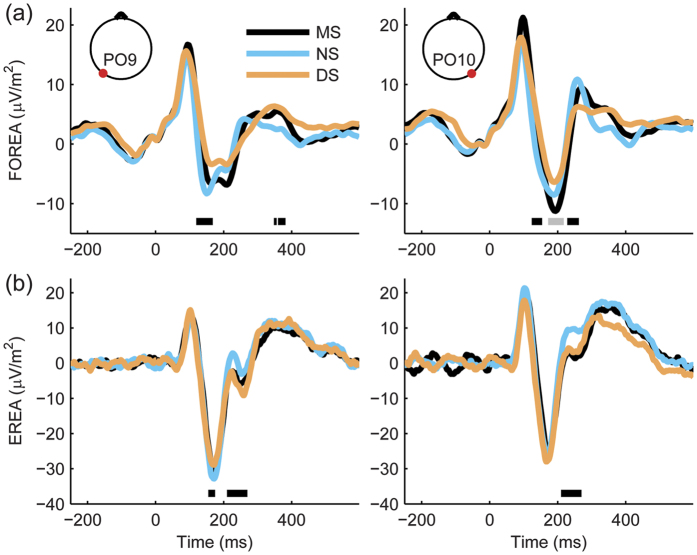
Grand average brain activity obtained during natural reading and word reading with fixed gaze. Fixation onset-related EEG activity (FOREA, panel (**a**)) and event-related EEG activity (EREA, panel (**b**)) are presented for the 3 letter spacing conditions (MS, NS and DS). To compare brain activity during natural reading (FOREA) and word reading with fixed gaze (EREA), left (PO9, left column) and right (PO10, right column) occipito-temporal channels were selected. Both FOREA and EREA averages were calculated using cleaned and scalp current density transformed single-trial EEG data. Effects of eye-tracking measures were regressed out from FOREA trials. In panel (**a**) horizontal lines mark time intervals with significant expertise-driven configural (black) and visual processing load (grey) effects (see Fig. 5 and Supplementary Fig. S4), while in panel (**b**) horizontal black lines denote time ranges selected for statistical assessment of letter spacing effects. Figure insets denote the location of PO9 and PO10 electrodes. To assess the potential effects of eye-movement properties on FOREA, grand average FOREA trends corresponding to significant expertise-driven configural and visual processing load effects (panel (**a**)) were also examined by averaging trials with current saccade amplitude falling into the [2.5 3]° range (Supplementary Fig. S6) and the overall quality of artefact elimination was also inspected (Supplementary Fig. S7).

We performed a control experiment (see Supplementary Methods, Control experiment 2) to test whether the expertise-driven configural and visual processing load effects on visual processing observed in the case of natural reading would also be reflected in brain activity during reading words presented at fixation. Here we restrict the comparison of natural and fixed-gaze word reading conditions to left and right occipito-temporal regions. Visual inspection of EEG activity time courses of channels PO9 and PO10 (Fig. 7) suggests that some of the effects obtained for natural reading may be also present in the case of word reading with fixed gaze, even though these effects may be less pronounced and have slightly different spatio-temporal characteristics. In an early time window ranging from 155 ms to 175 ms – possibly corresponding to the left-lateralized early expertise-driven configural effect in natural reading – there was a trend of more negative EEG activity in the case of NS as compared to the altered spacing conditions, however, the statistical analysis failed to reveal a significant main effect of letter spacing in this time interval (one-way repeated measures ANOVA (F(2, 32) = 2.09, p = 0.14; mean (M) and standard error of mean (SEM): M_MS_ = −27.77, SEM_MS_ = 5.30, M_NS_ = −31.03, SEM_NS_ = 5.70, M_DS_ = −27.67, SEM_DS_ = 4.80). In fact, the only significant effect was found in a later 210–270 ms interval, where the analysis using a two-way repeated measures ANOVA revealed a significant main effect for letter spacing (F(2, 32) = 9.29, p = 0.0006; while the main effect of electrode and the interaction between letter spacing and electrode were not significant: F(1, 16) = 3.77, p = 0.07; F(2, 32) = 1.37, p = 0.27, respectively), which might correspond to expertise-driven configural effects found in the natural reading condition in a similar time window. This is supported by the results of post hoc testing of letter spacing, showing a significantly higher EEG activity for the NS as compared to MS (p_Tukey_ = 0.0025) and DS conditions (p_Tukey_ = 0.0019). EEG activity of MS and DS conditions did not differ significantly (p_Tukey_ = 0.99). These results suggest that our approach developed to investigate visual processing during natural reading may provide additional information for identification and characterization of neural components of orthographic processing when compared to the traditional methods used to study word reading with fixed gaze.

## Discussion

Our results revealed that altering configural properties of the written text by modifying inter-letter spacing provides an efficient way to investigate the neural processes of visual information processing during natural reading. Expertise-driven configural effects were present in the fixation onset-related EEG activity in three consecutive time windows: 120–175 ms, 230–265 ms and 345–380 ms. The temporal profile of these configural effects found during natural reading is in a remarkable agreement with the results of previous studies^9^,^19^,^73^,^74^,^75^,^76^,^77^ investigating the temporal dynamics of the core components of visual word processing with fixed gaze. Furthermore, our results also revealed that the neural mechanisms that are engaged to handle the increased visual processing load as a result of enhanced crowding in case of decreasing letter spacing are reflected in right hemisphere lateralized occipito-temporal and parietal increase of EEG activity between 155–220 ms after fixation onset. The results of the current study are further strengthened by the fact that they were obtained with a data-driven analysis approach, without defining time windows of interest or constraining data analysis by waveform morphology (e.g. component peaks).

Models of visual word recognition proposed hierarchically organized stages of orthographic processing enabling the extraction of increasingly invariant and complex representations of written words^5^,^6^,^9^. At the low level orthographic processing stage, letter identity representations are computed within the first 200 ms after stimulus onset, which according to previous EEG studies using masked priming^73^,^74^,^75^,^76^,^77^ are already size invariant, but still position-sensitive, case-sensitive, and font-sensitive. This is followed by the computation of a more complex orthographic code, involving feature-invariant, abstract letter and word-form representations, taking place in the 200–300 ms time window^19^,^73^,^74^,^75^,^77^. In agreement with the scheme of visual word processing outlined above, neuroimaging studies^10^,^11^ showed that word recognition is subserved by the left fusiform gyrus, where orthographic representations are organized in a posterior-to-anterior hierarchy. Letter selective responses were revealed in a posterior part of the fusiform gyrus, a region called visual letter-form area^19^. Whereas, word-form selective responses were found in an adjacent, more anterior region of the fusiform gyrus, in the visual word-form area^10^,^11^, which computes a structural representation of the visual word as an ordered sequence of abstract letter identities. Furthermore, a recent study^19^ using magnetoencephalography (MEG) and intracranial recordings of local field potentials also provided direct support for the proposed dynamics of orthographic processing by showing that letter processing (identified by contrasting consonant strings vs. false fonts) occurs starting from ∼160 ms after stimulus onset, whereas word processing (identified by contrasting real words versus consonant strings) occurs starting from ∼225 ms.

Our results revealed that orthographic processing during natural reading involves sequential stages of information processing with remarkably similar temporal dynamics to those proposed by models of visual word processing with fixed gaze^6^,^9^. Altering the configural properties of the written text by changing the inter-letter spacing led to diminished EEG activity in the 120–175 ms and 230–265 ms time windows following fixation onset, most likely corresponding to the proposed low-level spatial and higher-level, abstract word-form selective orthographic processing stages, respectively. These results are in agreement with the expected effect of modifying letter spacing based on models of word recognition^6^,^9^: altering letter spacing would compromise both the parallel extraction of position-specific letter identity information as well as the subsequent computation of abstract letter combinations, such as bigrams and thus it is expected to affect both early letter coding and later word-form computation stages of orthographic processing. Our results also revealed a third component of expertise-driven configural effects that was present in a late time window ranging from 345 ms to 380 ms. Previous research on word reading with fixed gaze has found that EEG activity in a corresponding time window after stimulus presentation is sensitive to higher level whole-word processing^73^,^75^,^77^, which provides a parsimonious explanation for our results, since altering letter spacing might be expected to interfere with whole-word processing. Furthermore, by analysing how decreasing letter spacing affects fixation onset-related EEG activity across the spacing conditions we were able to identify the EEG signatures of the neural processes engaged to handle increased visual processing load during natural reading. Letter spacing affects the amount of visual information that is sampled and processed within a single fixation^28^. Overall visual processing load is increased with decreasing the letter spacing and this can clearly be seen in our eye movement results, showing prolongation of fixation durations with the reduction of letter spacing. Our findings revealed that EEG activity is increasing with the decrease of letter spacing in a time interval between 155–220 ms and thus suggest that increased visual processing demands will engage additional neural resources immediately after the early stage of orthographic processing. This appears to be in agreement with the results of previous research on object recognition with fixed gaze^78^,^79^ showing that increased processing load in the case of deteriorated, noisy images (e.g. faces) is reflected in the enhanced amplitudes of a positive going wave, the P2 component of the EEG responses peaking around 200 ms. Taken together, these results show that the methodological approach developed in the current study provides an efficient way of studying visual processing during natural reading. Our findings also imply that the time course of orthographic processing during natural reading might be remarkably similar to that found during word reading with fixed gaze. This conclusion is in agreement with recent intracranial EEG results^80^ showing similar object selective (including word selective) response dynamics in the human occipito-temporal cortex during a free viewing visual search task and static visual stimulation.

Fluent reading comes as a result of extensive practice. Reading skills develop gradually from childhood to adolescence^2^,^16^ and acquiring reading expertise has an overall effect on visual information processing^17^,^18^. Therefore, uncovering the neural processes which underlie reading expertise and enable the complex orthographic processing in natural reading to be executed by the human brain in a remarkably efficient and seemingly effortless way is of primary importance. Our results revealed that expertise adapts orthographic processing during natural reading to the second-order configural relations^25^ within the written words, i.e. the spatial distances among the letters in the case of text with normal spacing. Altering second order configural properties, independently of whether it is done by increasing or decreasing letter spacing will hamper both early and later stages of orthographic processing, as indicated by the reduced EEG activity during fixations. Furthermore, the strength of the expertise-driven configural EEG effects in the later time window showed a significant correlation with the participants’ reading speed, suggesting that our approach is sensitive enough to detect individual differences in the orthographic processing efficiency of skilled readers. Importantly, the expertise-driven configural effects were revealed by contrasting the fixation onset-related EEG activity during reading a text with normal letter spacing to that with increased and reduced spacing, and thus cannot be explained by the changes in low-level stimulus properties (e.g. spatial frequency, contrast, etc.) or in the strength of visual letter crowding^28^,^29^,^30^ as a result of spacing manipulation.

The earliest expertise-driven configural EEG effects were found in the 120–175 ms time window and were present bilaterally with a stronger magnitude over the left hemisphere. This is in agreement with previous results^20^,^21^,^22^ showing that in the case of reading words with fixed gaze, ERP responses in a similar time window – peaking in the well-known N1 component – are increasing in amplitude and become more left-hemisphere lateralized with reading skills. Furthermore, previous research on the effect of expertise on visual object processing has revealed that becoming an expert in processing a visual object category will lead to increased sensitivity to its configural properties^27^,^31^,^32^,^33^, which in turn is reflected in the N170 component^34^,^35^,^36^. Thus, the first configural EEG effect found in the present study might reflect expertise-driven modulation of early orthographic processing, involving parallel extraction of position-specific letter identity information as well as their combination into bigrams, which according to a recent study^77^ investigating word reading with fixed gaze might start already in this early time period. The next time window where EEG activity in the normal spacing condition was found to differ from the altered spacing conditions was between 230–265 ms, suggesting that it might be associated with the word-form processing stage of orthographic processing^19^,^73^,^74^,^75^,^77^. This later expertise-driven configural EEG effect was clearly present over both hemispheres, but reached the significance level only on the right side. At first glance, the right hemisphere lateralization of this effect appears to be inconsistent with the results of previous research providing converging evidence^10^,^11^ for a close association between word-form processing and a region of the left fusiform gyrus, the visual word form area. However, there is increasing evidence^81^ that despite the dominance of left hemisphere in visual word-form processing, the right hemisphere might play an important role in processing of orthographically irregular items that place more demands on visuospatial analysis. Previous research using masked priming and repetition paradigms suggest that the neural signatures of orthographic processing in the right hemisphere are most pronounced during the epoch of the N250 component of the ERP responses^73^,^74^,^75^. Based on these findings, our results might be interpreted as evidence that expertise optimizes visuospatial processes of word-form analysis in the right hemisphere to the text with normal letter spacing. Altering letter spacing impairs these processes and is reflected in decreased EEG activity in a time interval from 230–265 ms after fixation onset. The third component of the expertise-driven configural EEG effects was found over the left hemisphere in a time window ranging from 345 to 380 ms, most probably reflecting expertise effects present at the higher level whole-word processing stage^73^,^75^,^77^. Importantly, strength of the two later configural EEG effects was closely associated with participants’ individual reading speed, which strongly supports their behavioural relevance.

Although the SCD transform reduces the effects of volume conduction and provides more focal EEG activity, it does not substitute the solution of the inverse problem. Accordingly, our results can be interpreted only in the sensor space. To relate the obtained effects to specific brain regions, EEG recordings would have to be supplemented with individual MR anatomy and electrode locations for precise source reconstruction. Combination of EEG with other neuroimaging modalities such as MEG and fMRI could also contribute to the localization of neural sources corresponding to letters spacing effects.

The present study was aimed at investigating the visual processing during natural reading. Accordingly our analysis and discussion were focused on results found on posterior electrodes. Nevertheless, it is important to note that significant expertise-driven configural EEG effects were also found on centro-parietal and frontal electrodes with dynamics similar to that described for the occipito-temporal electrodes. Further studies are required to determine whether the effects found on the centro-parietal and frontal electrodes can be accounted for by the same neural sources as those found on the posterior channels, or alternatively, they are signatures of expertise effects on top-down modulation of orthographic processing^12^.

The hierarchical linear modelling approach allows for testing the linear effects of categorical and continuous experimental variables and eye-movement covariates. For even more sophisticated handling of differential overlap of fixation onset-related EEG activity novel deconvolution methods and tools would have to be developed and thoroughly validated^13^,^82^, and other approaches relying on general linear modelling could be also considered^54^. Moreover, for testing of potential non-linear effects non-linear models would also have to be applied^83^. By increasing the number of letter spacing levels, controlling for more eye-movement covariates and adding other visual experimental factors more detailed and even more reliable assessment of letters spacing effects as well as their interaction with other visual factors could be obtained.

The experiment was designed in a way to prevent the psycholinguistic variables from confounding the experimental factor of particular interest. To allow for continuous reading of whole paragraphs, the eye-tracking system was re-calibrated only if significant head movements were performed. Thus the available eye-tracking recordings are appropriate for assessment of eye-movement properties, but are not suitable for estimation of absolute gaze position with high precision. Potential effects of psycholinguistic variables and their interaction with letter spacing during natural reading would be of special interest. However, to test these effects more constrained experiments with frequent testing and re-calibration of the eye-tracking system would have to be carried out.

The importance of understanding the neural basis of orthographic processing during natural reading is further strengthened by recent findings showing that inefficient visual processing might contribute to the poor reading abilities of dyslexics^39^. It was found that increasing letter spacing significantly improved reading efficiency in dyslexic children, but had no effect in children with normal reading skills. These results suggest that dyslexics might be unable to handle the high visual processing demand even in the case of text with normal letter spacing. However, to what extent impaired orthographic processing in dyslexia can be accounted for by an existing visual information processing deficit, lack of expertise or both, remains an important open question. The methodological approach presented in the current study seems to be appropriate to address this question, since it allows for the measurement of both the individual level of expertise in orthographic processing as well as the ability to handle increased visual processing load during natural reading.

## Additional Information

**How to cite this article**: Weiss, B. *et al*. Visual processing during natural reading. *Sci. Rep.* **6**, 26902; doi: 10.1038/srep26902 (2016).

## Supplementary Material

Supplementary Information
