# Supplementary material for: Visual processing during natural reading
Source: Sci Rep. 2016 May 27;6:26902. doi: 10.1038/srep26902 (PMC4882504; doi:10.1038/srep26902)
Supplement: Supplementary Information [file srep26902-s1.doc]

# Supplementary Information

**Visual processing during natural reading**

**Béla Weiss^1,*^, Balázs Knakker^1,2^, Zoltán Vidnyánszky^1,3,*^**

^1^ Brain Imaging Centre, Research Centre for Natural Sciences, Hungarian Academy of Sciences, Budapest 1117, Hungary

^2^ Faculty of Information Technology and Bionics, Pázmány Péter Catholic University, Budapest 1083, Hungary

^3^ Department of Cognitive Science, Budapest University of Technology and Economics, Budapest 1111, Hungary

^*^ Corresponding authors: Béla Weiss (e-mail: weiss.bela@ttk.mta.hu; phone: +36 1 3826904; address: Magyar tudósok körútja 2, Budapest 1117, Hungary), Zoltán Vidnyánszky (e-mail: vidnyanszky.zoltan@ttk.mta.hu; phone: +36 1 3826905; address: Magyar tudósok körútja 2, Budapest 1117, Hungary)

# Supplementary Methods

## Control experiment 1 – One-minute reading

### Participants

Twenty-nine undergraduate students (19 female) participated in this control experiment. Average age was 22.72 years (SD=2.2 years, range: 20-28 years). The sample consisted of 24 right-handed, 3 left-handed and 2 ambidextrous subjects, as assessed by the standard Edinburgh Handedness Inventory. All of them were native speakers of Hungarian, reported having typical reading skills and had normal or corrected-to-normal vision. None of them had any history of neurological or psychiatric diseases. The experiment was approved by the local ethics committee of the Department of Cognitive Science, Budapest University of Technology and all methods were carried out in accordance with the approved guidelines. Subjects gave written informed consent.

### Visual stimuli and experimental procedure

For this control experiment 12 OECD PISA texts were selected from those used in the main experiment and presented with the same settings. Besides MS, NS and DS conditions, a fourth condition was also considered to test the potential effects of more pervasive configural text modulations on reading speed. For this purpose vertical (V) text lines were generated by rotating horizontal text lines with normal spacing by 90° counterclockwise. Horizontal text was read from left to right, while vertical text lines were read in the bottom-top direction. A margin of 2 cm was applied on right and left (horizontal text lines) or lower and upper (vertical text lines) borders of the white background. In contrast to the main experiment, where the letter spacing of consecutive text lines changed in a pseudo-random order, here the same letter spacing and text orientation was kept within a paragraph.

Paragraphs were organized into 3 blocks. During the 1^st^ block, 4 paragraphs were presented using MS, NS, DS and V conditions. Subjects were instructed to read the whole text at their own pace. The 2^nd^ block also contained 4 texts with the same conditions as in block 1. However, in this case, only 1 minute was allocated for each paragraph, after which the currently presented text line disappeared. Subjects had to read texts silently at their own pace and write down for all paragraphs the last 2 words they read. The 3^rd^ block was very similar to the 2^nd^ one except that subjects had to read aloud. The order of paragraphs and conditions was randomized.

Generation of stimuli, control of the experimental procedure and collection of subjects’ responses were performed using custom written scripts and the Psychophysics Toolbox 3 ^1–3^ under MATLAB R2008a (The MathWorks Inc., Natick, MA, USA).

### Data analysis

Here we show the results for one-minute silent reading only, more detailed presentation of results is to be available elsewhere. Reading speed was defined as the ratio of the number of words that have been read and the reading time. Effects of letter spacing on reading speed were assessed by repeated measures ANOVA with one factor having 3 levels (MS, NS and DS) and Tukey’s Honest Significant Difference (HSD) post hoc testing. A paired t-test was used to evaluate the effect of text orientation on reading speed by comparing conditions NS and V. Normality and sphericity assumptions were checked by the Shapiro-Wilk W and the Mauchly tests, respectively. Finally, the correlations between reading speed of the NS and the other three conditions were analysed. Correlation coefficients were calculated with the same settings as in the case of the main natural reading experiment, except for the levels of significance and confidence, that were corrected according to the total number (N=3) of tested correlations (p_Cor_<0.05, 98.33 % CI).

## Control experiment 2 – Word reading with fixed gaze

### Participants

Nineteen healthy right-handed young adults participated in this study. Two of them had insufficient number of artefact-free data segments and accordingly only 17 subjects (11 female; mean±SD age: 24±2.10 years) entered statistical analysis. All of them were native speakers of Hungarian, reported having typical reading skills and had normal or corrected-to-normal vision. None of them had any history of neurological or psychiatric diseases. The experiment was approved by the local ethics committee of the Department of Cognitive Science, Budapest University of Technology and all methods were carried out in accordance with the approved guidelines. Participants gave informed consent before the beginning of the measurements.

### Visual stimuli and experimental procedure

The stimuli were 4 and 5 letter Hungarian nouns from two semantic categories (living and non-living), presented centrally on a 26” liquid-crystal display using a monospace font (Courier New). Random flanker words were also presented on both sides of the central word, to mimic the visual context during natural reading. There were three presentation conditions corresponding to the 3 letter spacing conditions in the main experiment (MS, NS and DS). Normal words subtended approximately 2° in the horizontal dimension. A small blue fixation dot was always present in the centre of the screen. The background was white. The subjects were seated in a dimly lit room, their head was supported by a chin rest in a distance of 56 cm from the screen. The experiments were conducted in 6 runs, each lasting cca. 8 minutes, with some minutes of rest in between. Within runs, letter spacing of the words was constant. Condition order was counterbalanced across subjects. In half of the trials, words were presented without flanking words – these trials are not included in this study.

In each trial, a word was presented for 800 milliseconds. Subjects were told to respond after stimulus offset with a mouse button press, indicating which category the word (living or non-living) they had seen belonged to. The response interval was maximized in 2 seconds. The length of the inter-trial- interval (ITI, starting from the time of the response or from the end of the response interval) was chosen from a uniform probability distribution between 1250 and 1750 milliseconds. After every third trial an additional 650 ms of pause was added with the fixation dot turning red, and the subjects were asked to try to blink only during this period. The frequency and length of these blink windows were sometimes adjusted to the given subject’s propensity to blink. Stimulus presentation and subject response registration was implemented in MATLAB R2008a (The MathWorks Inc., Natick, MA, USA) using PsychToolbox version 3 ^1–3^.

### Recordings

EEG was acquired using 62 electrodes (Brain Products actiCAP; amplifier: BrainAmp Standard; Brain Products GmbH, Munich, Germany) mounted on an elastic cap according to the 10-10 system. Electrooculogram (EOG) activity was monitored by 3 EEG channels and an additional electrode that was placed below the right eye. The sampling rate was 500 Hz. Eye movements were recorded from participants’ left eye using iView X™ Hi-Speed 1250 system (SensoMotoric Instruments GmbH, Teltow, Germany) at a sampling rate of 1250 Hz.

### Data analysis

Pre-processing of the EEG signal was done in Brain Vision Analyzer. The signal was band-pass filtered (Butterworth zero-phase filter, 0.5-35 Hz, 12 dB/octave) and segmented. Segments containing artefacts were marked using amplitude ([-50 50] µV for EOG, [-80 80] µV for EEG channels), amplitude difference (80 µV for EOG, 120 µV for EEG channels) and voltage step thresholds (10 µV per sample) and by visual inspection; these segments were not used in further analyses. Data were imported to MATLAB, and surface Laplacian approximations of the scalp current density was calculated using the CSD Toolbox ^4^ (unit sphere radius, m=4, λ=10^-5^, the maximum degree of Legendre polynomials was set to 10). Artefact-free segments were baseline-corrected ([-200 0] ms) and averages for conditions of interest were computed for each subject. Channels PO9 and PO10 were used in statistical analyses. Time ranges of [155 175] ms and [210 270] ms were selected based on group-level results of the main experiment and visual inspection of EEG amplitude trends. EEG amplitudes within these time intervals were averaged and entered repeated measures ANOVA with Tukey’s HSD post hoc testing. The [155 175] ms time range was assessed by a one-way ANOVA test (factor spacing with levels MS, NS and DS) considering the PO9 electrode only, while a two-way ANOVA (factors: spacing with levels MS, NS and DS; electrode with levels PO9 and PO10) was used for the [210 270] ms time window. Assumptions of normality and sphericity were fulfilled as assessed by the Shapiro-Wilk W and the Mauchly tests, respectively. Statistical analysis was carried out in STATISTICA (StatSoft Inc., Tulsa, OK, USA).

# Supplementary References

1. Brainard, D. The Psychophysics Toolbox. *Spat. Vis.* **10,** 433–436 (1997).

2. Pelli, D. G. The VideoToolbox software for visual psychophysics: transforming numbers into movies. *Spat. Vis.* **10,** 437–442 (1997).

3. Kleiner, M., Brainard, D. & Pelli, D. What’s new in Psychtoolbox-3? *Perception* **36,** (2007).

4. Kayser, J. & Tenke, C. E. Principal components analysis of Laplacian waveforms as a generic method for identifying ERP generator patterns: I. Evaluation with auditory oddball tasks. *Clin. Neurophysiol.* **117,** 348–368 (2006).

# Supplementary Figures


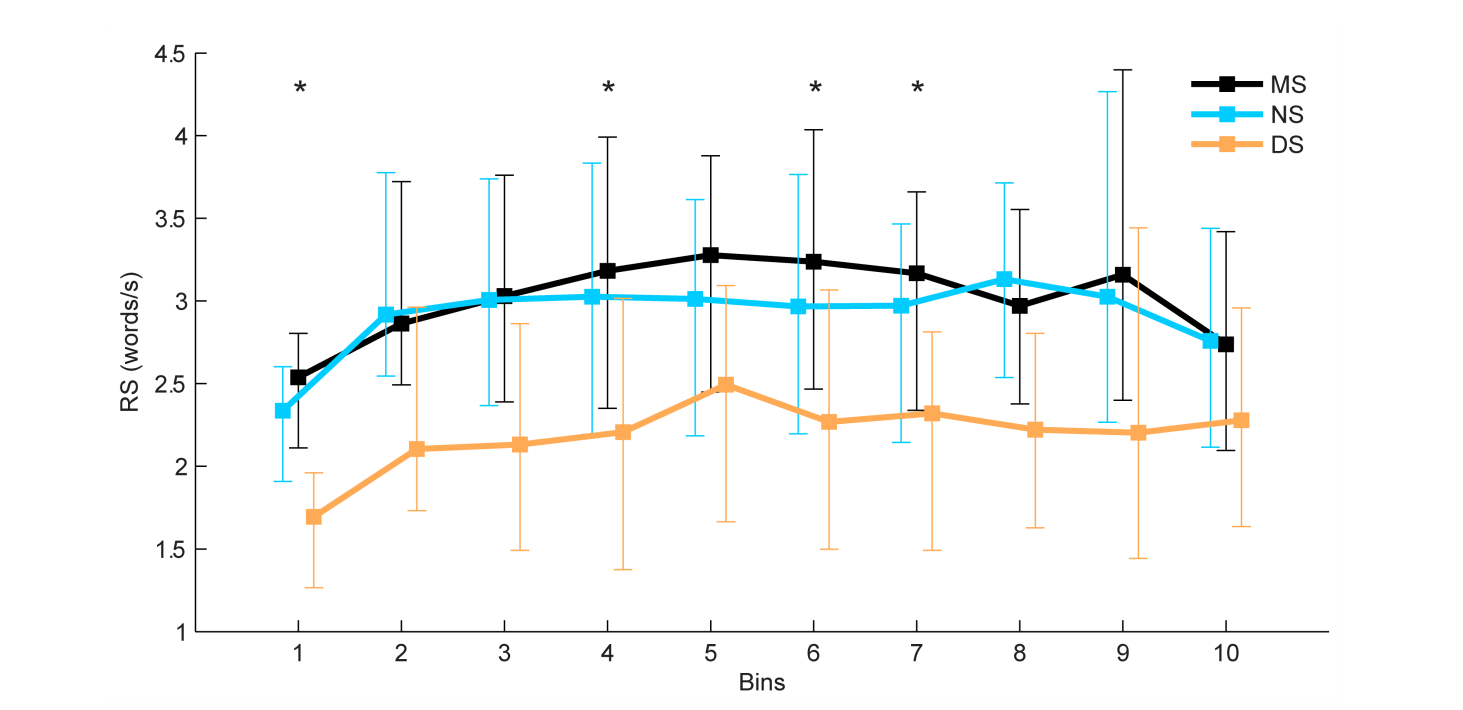


Supplementary Figure S1. The time course of reading speed during the natural reading experiment.

For all the 3 letter spacing conditions (MS, NS and DS) data was divided into 10 temporally consecutive bins with equal amounts of text in them, and individual median reading speed (RS) was calculated. The square marks on the error bars denote group-level median RS values. The lower and upper whiskers mark 25^th^ and 75^th^ percentiles, respectively. Here our main aim was to reveal the background of the reading speed difference between the MS and NS conditions obtained using pooled data (see Fig. 3). To assess the time course of reading speed differences between the MS and NS conditions, the Wilcoxon matched pairs test was applied for all bins. Significantly (p<0.05) faster reading was found for the MS as compared to the NS condition in several bins with a predominance of middle ones (bins 4-7, marked by asterisks at the top of the figure) suggesting that this difference evolves with practicing. However, after Bonferroni correction no significant differences remained. Although the differences of reading speed for the other 2 pairs of letter spacing conditions were statistically not evaluated by means of hypothesis testing, a trend of slower reading was observed for the DS condition as compared to MS and NS conditions across all bins.


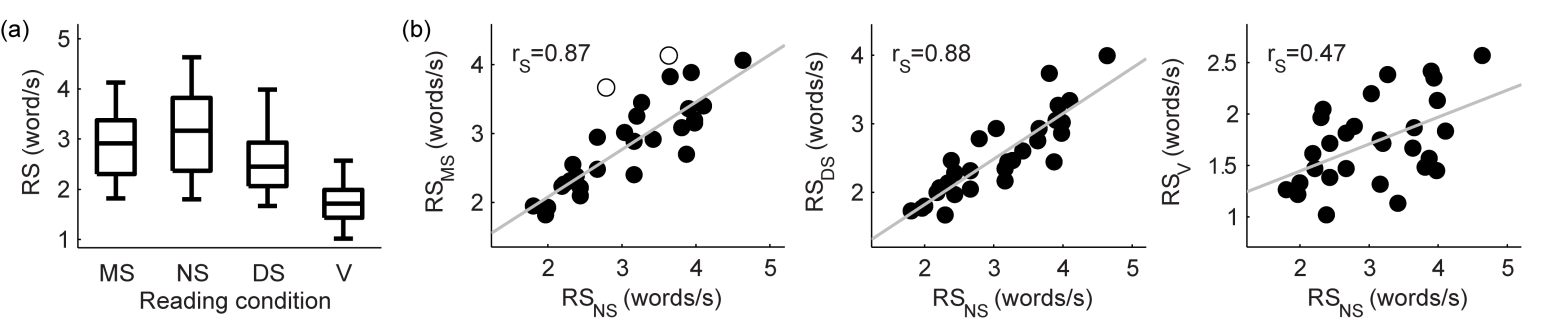


Supplementary Figure S2. Reading speed results obtained for the one-minute silent reading experiment.

Panel (a) shows box and whisker plots of reading speed (RS) for 4 reading conditions (horizontal text orientation with minimal (MS), normal (NS) and double letter spacing (DS); vertical text orientation with normal letter spacing (V)) based on individual median values. Whiskers represent minima and maxima. It was found that letter spacing has a significant effect on reading speed (one-way repeated measures ANOVA main effect F(2,56)=24.67, p<0.00001); Tukey’s HSD post hoc results: RS_NS_>RS_DS_ and RS_MS_>RS_DS_, p_Tukey_<0.001; RS_NS_>RS_MS_, p_Tukey_=0.054). Considering the effect of text orientation, reading was faster for the horizontally presented text (NS) as compared to the vertical condition (V), t(28)=10.7, p<0.00001. Scatter plots in panel (b) present the relationship between reading speed of the NS and the other 3 reading conditions (MS, DS and V). Significant (p_Cor_<0.05) positive correlations were found between the reading speed of the NS and the other 2 horizontal conditions (RS_NS_ vs. RS_MS_: r_S_=0.87, 98.33% CI=[0.66 0.94], NO=2; RS_NS_ vs. RS_DS_: r_S_=0.88, CI=[0.70 0.96]). A positive correlation was also found between the reading speed of horizontal and vertical conditions with normal inter-letter spacing, however, the level of significance was not reached in this case (r_S_=0.47, CI=[-0.01 0.78]). Grey lines denote best fit lines, open circles mark bivariate outliers detected by the adjusted box-plot rule and r_S_ stands for the Spearman’s correlation coefficient.


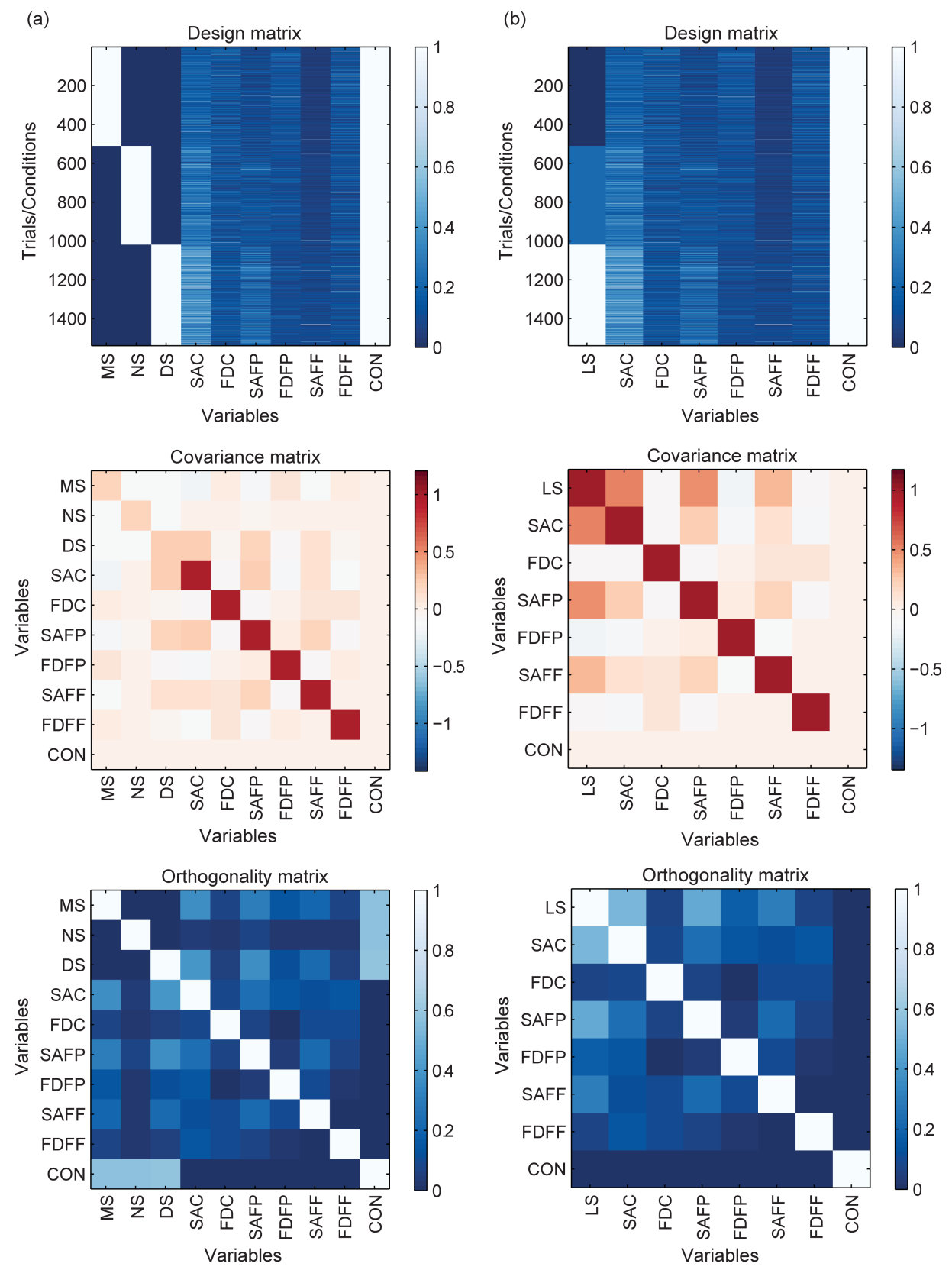


Supplementary Figure S3. Design, covariance and orthogonality matrices of subject #1.

Two different models were used at the subject level of hierarchical linear modelling analysis. In the analysis of covariance (ANCOVA) model (panel (a)) letter spacing (LS) was a factorial variable with 3 levels (MS, NS and DS), while in the case of the multiple linear regression (MLR) model LS was a continuous variable. To control for potential covariate effects of eye movements, 6 eye-movement variables (SAC, FDC, SAFP, FDFP, SAFF and FDFF) were included in both ANCOVA and MLR models. In the case of design matrices continuous regressors were normalized to the [0 1] interval to present continuous and factorial variables together using the same colour map. During analysis z-score normalization was applied on the continuous regressors. In concordance with the group-level results presented in Fig. 2, the sample design, covariance and orthogonality matrices of the MLR model indicate a positive correlation between the letter spacing and saccade amplitude variables. Accordingly, a proper handling of multicollinarity and validation of results should be realized. To validate the group-level letter spacing effects, we compared the grand average time course of fixation onset-related EEG activity (FOREA) generated by averaging all trials with the time course of grand average FOREA obtained by averaging only trials with a current saccade amplitude within a narrow range (see later Fig. 7 and Supplementary Fig. S6). Abbreviations: SAC – current saccade amplitude; FDC – current fixation duration; SAFP – first preceding saccade amplitude; FDFP – first preceding fixation duration; SAFF – first following saccade amplitude; FDFF – first following fixation duration; CON – constant term.


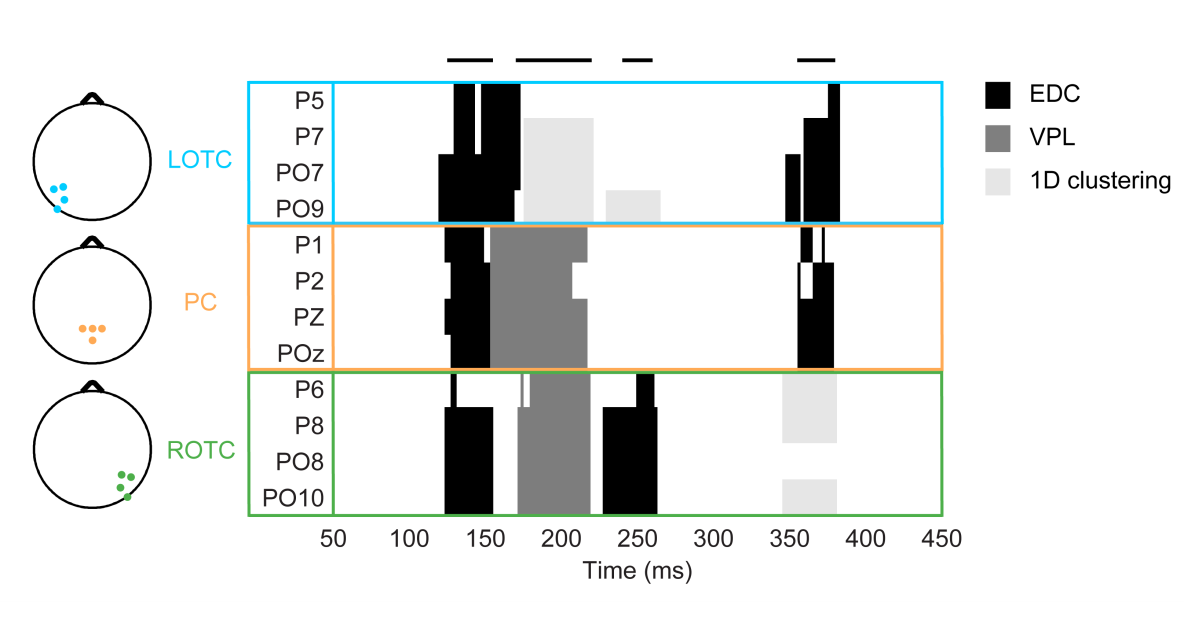


Supplementary Figure S4. Group-level significant expertise-driven configural and visual processing load effects for selected channel clusters.

In Fig. 5, significant expertise-driven configural (EDC) and visual processing load (VPL) effects with distinct spatio-temporal properties have been revealed. To assess the relationship between reading skill and significant effects of letter spacing modulation on brain activity, spatio-temporal clusters were defined for averaging of subject-level β coefficients. Based on the spatio-temporal distribution of significant EDC and VPL effects three channel clusters were generated: left occipito-temporal (LOTC; blue; channels P5, P7, PO7, PO9), parietal (PC; orange; channels P1, P2, Pz, POz) and right occipito-temporal (ROTC; green; channels P6, P8, PO8, PO10). Here, spatio-temporal samples with significant EDC and VPL effects are visualized by black and dark grey patches, respectively. Horizontal lines at the top of the figure mark the time intervals (125-155 ms, 170-220 ms, 240-260 ms and 355-380 ms) that were considered for further analysis. Selection of the time ranges was performed in a way to optimize the distribution of significant samples across the channel clusters as well as across the channels within the clusters. Only those combinations of channel clusters and time ranges entered correlation analysis that contained significant EDC or VPL effects. This approach resulted in 8 correlations between reading skill and the significant effects of letter spacing modulation. Significant results revealed by 1D clustering in time domain are presented by light grey patches. However, these effects were not taken into account during the generation of spatio-temporal clusters for correlation analysis. Permutation testing with 1D clustering was only used to validate visually observable trends that were not detected by spatio-temporal clustering presumably due to their limited spatial extent. Using 1D clustering significant EDC effects were found in the 235-265 ms (PO9) and 345-380 ms (P6, P8, PO10) time ranges, while a significant VPL effect was found in the LOTC cluster in the 175-220 ms time interval.


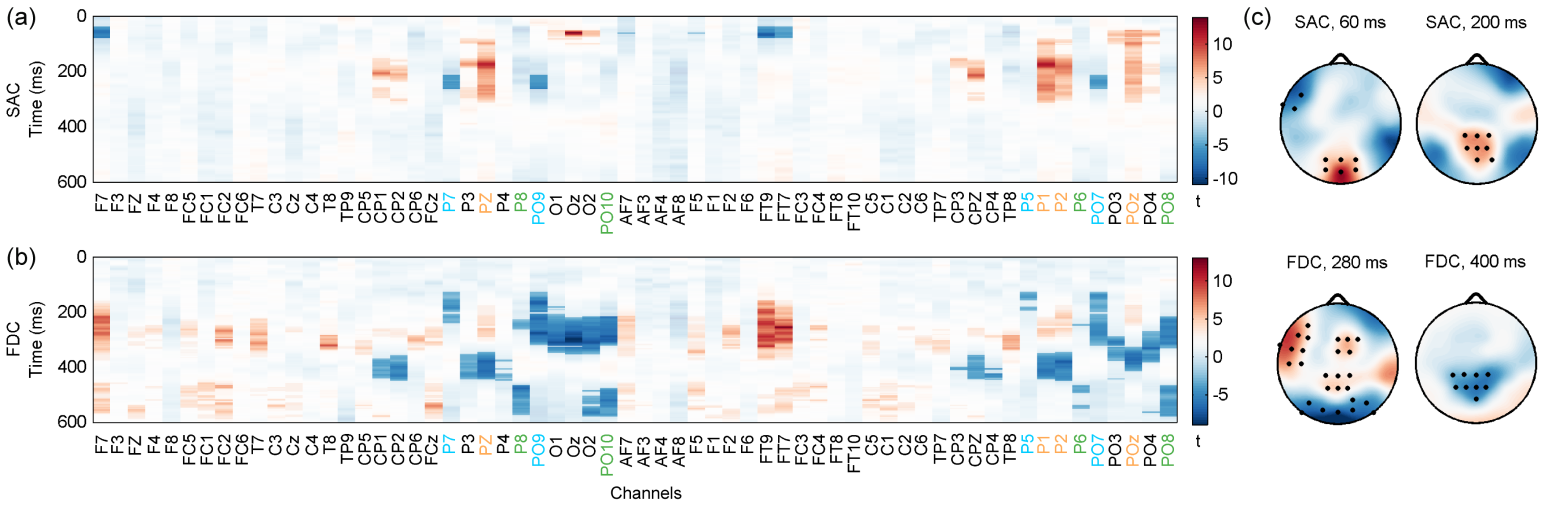


Supplementary Figure S5. Group-level statistical results of eye-tracking covariates obtained from the MLR model.

The figure presents spatio-temporal distributions of group-level t-values for current saccade amplitude (SAC) and current fixation duration (FDC) eye-tracking covariates (panels (a) and (b)) as well as topographic distributions of t-values for time instances of special interest (panel (c)). In panels (a) and (b) significant (p_Cluster_<0.05) t-values are fully opaque, while the opacity was reduced for the rest of the samples. In panel (c) significant (p_Cluster_<0.05) t-values are denoted by marking the corresponding channels, the colour scales correspond to the colour bars in panels (a) and (b). Significant (p_Cluster_<0.05) spatio-temporal clusters were obtained for both SAC and FDC covariates. In the case of SAC, the strongest effect was found in an early (50-75 ms) positive occipito-central cluster (channels O1, Oz, O2, PO3, POz, PO4) with the highest t-value at channel Oz around 60 ms. A latter positive cluster is present in the parieto-central region (channels CP1, CPz, CP2, CP3, P1, Pz, P2, P3, PO3, POz, PO4) with a longer duration (80-315ms). Several peaks can be found within this cluster including those around 100 ms (Pz, POz), 175 ms (P1, Pz, P2) and 210 ms (CP1, CPz). Considering significant (p_Cluster_<0.05) negative trends, beside the early transient (40-80 ms) effect in the left fronto-temporal region (channels AF7, F5, F7, FT7, FT9), a left occipito-temporal cluster (channels P7, PO7, PO9) is present in the 215-265 ms time interval. For FDC, the second covariate in the MLR model, clusters with larger spatio-temporal extent were revealed. The earliest significant (p_Cluster_<0.05) effects appear around 130 ms. This negative cluster starts in the left occipito-temporal region (channels P5, P7, PO7, PO9) and it expands to occipital and right occipito-temporal channels (O1, Oz, O2, P8, PO8, PO10) starting from about 210 ms. This effect lasts until around 330 ms and shifts into the anterior direction generating a parietal cluster (CP1, CP3, CPz, CP2, CP4, P1, P3, Pz, P2, P4, PO3, POz, PO4) in the 350-450 ms time range. At the end, a significant (p_Cluster_<0.05) negative cluster that mainly involves occipital and occipito-temporal channels (O2, P6, P8, PO8, PO10) is present in the 460-575 ms interval. The significant (p_Cluster_<0.05) positive cluster of the FDC covariate with highest t-values can be found in the left fronto-temporal region (strongest effects in channels F7, FT9, FT7) in the 160-375 ms time interval. Finally, smaller significant (p_Cluster_<0.05) positive fronto-central clusters appear around 270 ms and 540 ms.


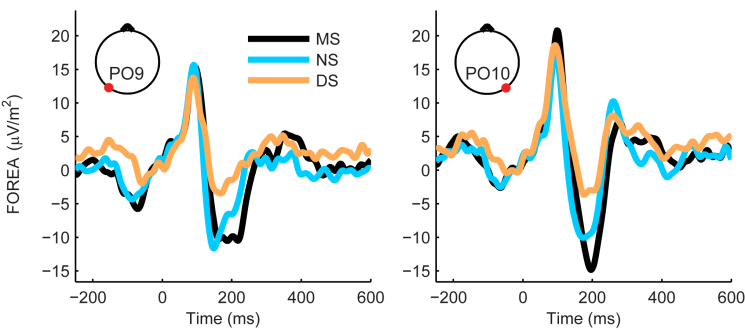


Supplementary Figure S6. Grand average fixation onset-related EEG activity obtained by averaging trials with constrained current saccade amplitudes.

To examine the potential effects of eye-tracking covariates on the fixation onset-related EEG activity (FOREA) trends that correspond to significant expertise-driven configural (EDC) and visual processing load (VPL) effects (Fig. 7 panel (a)), grand average FOREAs were also generated by matching the trials across the letter spacing conditions (MS, NS and DS) according to the incoming saccade amplitude. Only trials with current saccade amplitudes falling into the [2.5 3]° range were considered. FOREA averages were calculated using cleaned and scalp current density transformed single-trial EEG data. The [2.5 3]° range for the current saccade amplitude measure was selected to maximize the number of appropriate trials (mean=120.3, SD=59, range: 18-219, the number of subjects having less than 50 trials was 3). For each subject the number of trials was randomly balanced across the letter spacing conditions. It can be seen that FOREA trends corresponding to the EDC and VPL effects in Fig. 7 panel (a) are preserved in grand average FOREA with constrained current saccade amplitude. These results suggest that the significant EDC and VPL effects revealed in this study are not confounded by the current saccade amplitude, and accordingly support the assumption that the obtained effects have a neural origin. Figure insets denote the location of PO9 and PO10 electrodes.


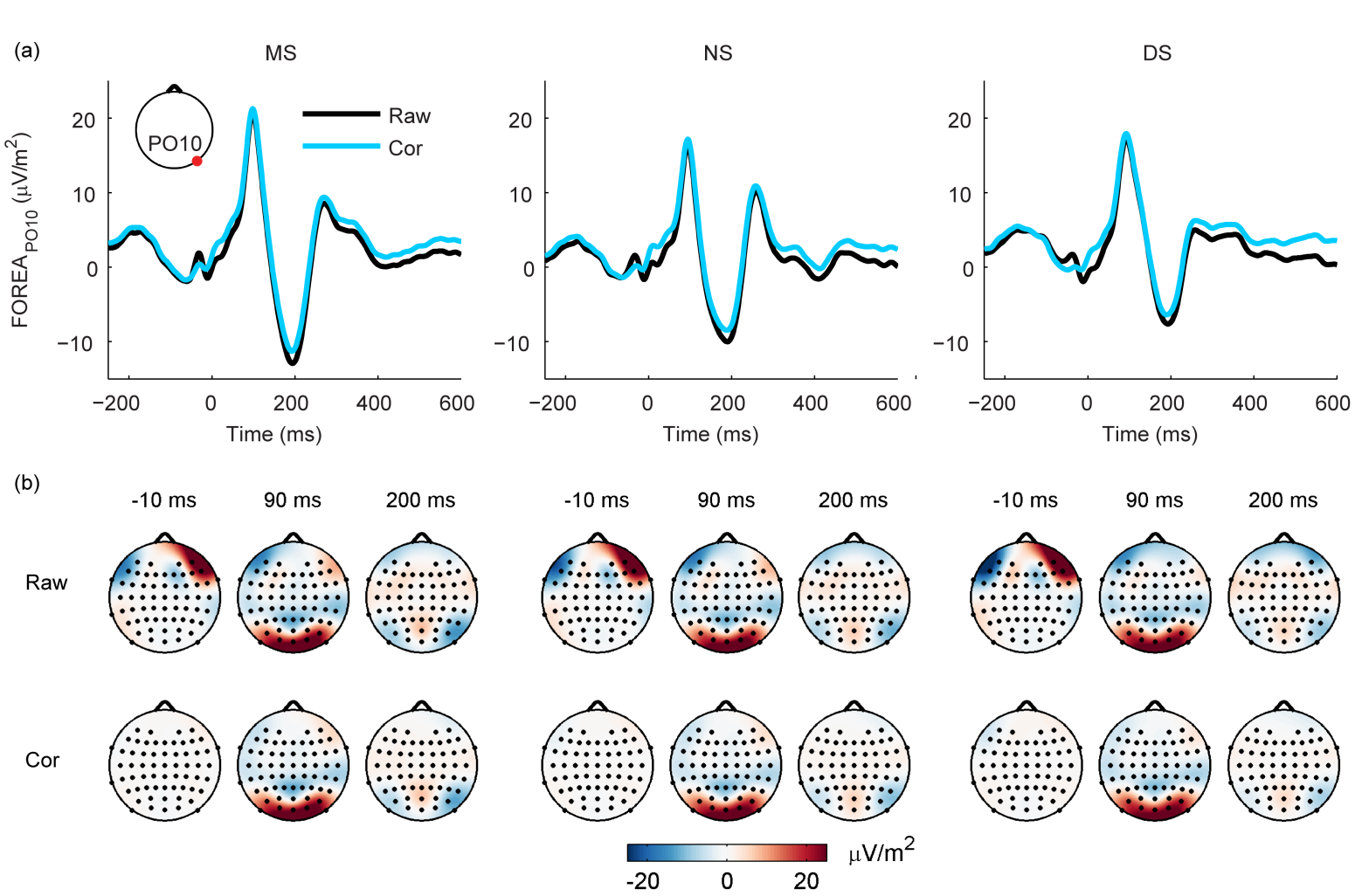


Supplementary Figure S7. Artefact elimination results.

Time course of raw (Raw) and artefact corrected (Cor) grand averages of fixation onset-related EEG activity (FOREA) are presented for channel PO10 in panel (a), and topographic distributions of Raw and Cor FOREA are provided for specific time points of special interest (-10 ms, 90 ms and 200 ms) in panel (b). Raw and Cor results were obtained using the same trials and pre-processing steps, except for the eye movement artefact elimination procedure that was only applied in the case of Cor. All trials were scalp current density transformed before averaging. The results demonstrate that our ICA-based artefact elimination procedure that is enhanced by eye-tracking information successfully eliminates even large frontal eye movement artefacts and preserves EEG activity that is presumably of neural origin. Although no significant differences of artefact rejection effects can be observed across the letter spacing conditions (MS, NS and DS) by visual inspection, a hierarchical linear modelling approach should be applied for FOREA analysis to assure further elimination of residual artefacts by including eye-tracking regressors into the linear models. The similarity of FOREA trends before and after elimination of artefacts also suggests that the obtained effects are robust and are not artificially generated by the artefact elimination procedure. Locations of the 62 EEG channels are marked by black dots in panel (b).

# Supplementary Tables

Supplementary Table S1. The list of abbreviations and symbols.

| **Abbreviations and symbols** | **Description** |
| --- | --- |
| **1D clustering** | cluster-based permutation test applied at group level by performing clustering only along the time dimension |
| **ANCOVA** | analysis of covariance |
| **ANCOVA_LSCONT_** | the -1 2 -1 contrast defined on the MS, NS and DS levels of the letter spacing variable of the ANCOVA model; it is used to test the potential expertise-driven configural effects of letter spacing on fixation onset-related EEG activity |
| **ANOVA** | analysis of variance |
| **C** | current; used to denote the current fixation durations and the corresponding current (also called incoming) saccade amplitudes |
| **CI** | bootstrap confidence intervals |
| **CON** | constant term used in ANCOVA and MLR models |
| **CONT** | contrast; refers to the ANCOVA_LSCONT_ contrast used to test the expertise-driven configural effects of letter spacing; see also ANCOVA_LSCONT_ |
| **dh** | dh parameter of threshold-free cluster enhancement |
| **DS** | double spacing |
| **E** | cluster extent exponent used for threshold-free cluster enhancement |
| **EDC** | expertise-driven configural |
| **EEG** | electroencephalogram |
| **EOG** | electrooculogram |
| **EREA** | event-related EEG activity; used to denote EEG activity obtained during the fixed-gaze word reading experiment |
| **ERP** | event-related potential |
| **F** | F-statitic of parametric ANOVA tests |
| **FD** | fixation duration |
| **FF** | first following; used to denote first saccade amplitudes and fixations durations that follow the current ones |
| **fMRI** | functional magnetic resonance imaging |
| **FOREA** | fixation onset-related EEG activity |
| **FP** | first preceding; used to denote first saccade amplitudes and fixations durations that precede the current ones |
| **H** | threshold exponent used for threshold-free cluster enhancement |
| **HSD** | refers to the Tukey’s Honest Significant Difference post hoc test |
| **ICA** | independent component analysis |
| **LOTC** | left occipito-temporal channel cluster; channels P5, P7, PO7, PO9 |
| **LS** | letter spacing |
| **m** | spline flexibility constant used for scalp current density transformation |
| **M** | mean |
| **MEG** | magnetoencephalography |
| **MLR** | multiple linear regression |
| **MS** | minimal spacing |
| **NO** | number of outliers |
| **NS** | normal spacing |
| **OECD** | Organisation for Economic Co-operation and Development |
| **p_Bonferroni_** | Bonferroni corrected p values |
| **PC** | parietal channel cluster; channels Pz, P1, P2, POz |
| **p_Cluster_** | p values obtained by cluster-based permutation testing |
| **p_Cor_** | corrected p values provided by the Robust Correlation Toolbox |
| **PISA** | Programme for International Student Assessment |
| **PT_1_** | initial saccade peak velocity detection threshold of the adaptive algorithm used for analysis of eye-tracking data |
| **PTMV** | percent of trials with missing values |
| **p_Tukey_** | p values obtained by the Tukey’s Honest Significant Difference post hoc test |
| **Q** | quality factor of the applied notch filter |
| **R^2^** | a standard R^2^ measure threshold used to detect EEG segments with abnormal linear trends, defines the minimal fit between the EEG data and a line of minimal slope |
| **ROTC** | right occipito-temporal channel cluster; channels P6, P8, PO8, PO10 |
| **r_S_** | skipped Spearman’s correlation coefficients |
| **RS_AVG_** | average reading speed |
| **SA** | saccade amplitude |
| **SCD** | scalp current density |
| **SD** | standard deviation |
| **SEM** | standard error of mean |
| **t** | t-statistic of t-tests |
| **TFCE** | threshold-free cluster enhancement |
| **V** | vertical; refers to reading of vertical text lines that were generated by rotating horizontal text lines with normal letter spacing by 90° counterclockwise |
| **VPL** | visual processing load |
| **α_AA_** | α parameter of the adaptive algorithm used for analysis of eye-tracking data |
| **β_AA_** | β parameter of the adaptive algorithm used for analysis of eye-tracking data |
| **β_ANCOVA-FDC_** | coefficient of the current fixation duration covariate of the ANCOVA model |
| **β_ANCOVA-FDFF_** | coefficient of the first following fixation duration covariate of the ANCOVA model |
| **β_ANCOVA-FDFP_** | coefficient of the first preceding fixation duration covariate of the ANCOVA model |
| **β_ANCOVA-LSCONT_** | coefficient of the ANCOVA_LSCONT_ contrast, it is used to test the potential expertise-driven configural effects of letter spacing on fixation onset-related EEG activity |
| **β_ANCOVA-SAC_** | coefficient of the current saccade amplitude covariate of the ANCOVA model |
| **β_ANCOVA-SAFF_** | coefficient of the first following saccade amplitude covariate of the ANCOVA model |
| **β_ANCOVA-SAFP_** | coefficient of the first preceding saccade amplitude covariate of the ANCOVA model |
| **β_AVG_** | average of β_MLR-LS_ or β_ANCOVA-LSCONT_ coefficients in predefined spatio-temporal clusters |
| **β_MLR-FDC_** | coefficient of the current fixation duration covariate of the MLR model |
| **β_MLR-FDFF_** | coefficient of the first following fixation duration covariate of the MLR model |
| **β_MLR-FDFP_** | coefficient of the first preceding fixation duration covariate of the MLR model |
| **β_MLR-LS_** | coefficient of the letter spacing variable of the MLR model; it is used to test the potential effects of visual processing load on fixation onset-related EEG activity |
| **β_MLR-SAC_** | coefficient of the current saccade amplitude covariate of the MLR model |
| **β_MLR-SAFF_** | coefficient of the first following saccade amplitude covariate of the MLR model |
| **β_MLR-SAFP_** | coefficient of the first preceding saccade amplitude covariate of the MLR model |
| **λ** | smoothing constant used for scalp current density transformation |
| **χ^2^** | χ^2^-statistic of the non-parametric Friedman ANOVA test |
